# Diversity and Composition of Demersal Fishes along a Depth Gradient Assessed by Baited Remote Underwater Stereo-Video

**DOI:** 10.1371/journal.pone.0048522

**Published:** 2012-10-31

**Authors:** Vincent Zintzen, Marti J. Anderson, Clive D. Roberts, Euan S. Harvey, Andrew L. Stewart, Carl D. Struthers

**Affiliations:** 1 Museum of New Zealand Te Papa Tongarewa, Wellington, New Zealand; 2 New Zealand Institute for Advanced Study, Massey University, Albany Campus, Auckland, New Zealand; 3 School of Plant Biology and University of Western Australia Oceans Institute, University of Western Australia, Crawley, Western Australia, Australia; Technical University of Denmark, Denmark

## Abstract

**Background:**

Continental slopes are among the steepest environmental gradients on earth. However, they still lack finer quantification and characterisation of their faunal diversity patterns for many parts of the world.

**Methodology/Principal Findings:**

Changes in fish community structure and diversity along a depth gradient from 50 to 1200 m were studied from replicated stereo baited remote underwater video deployments within each of seven depth zones at three locations in north-eastern New Zealand. Strong, but gradual turnover in the identities of species and community structure was observed with increasing depth. Species richness peaked in shallow depths, followed by a decrease beyond 100 m to a stable average value from 700 to 1200 m. Evenness increased to 700 m depth, followed by a decrease to 1200 m. Average taxonomic distinctness △^+^ response was unimodal with a peak at 300 m. The variation in taxonomic distinctness Λ^+^ first decreased sharply from 50 to 300 m, then increased beyond 500 m depth, indicating that species from deep samples belonged to more distant taxonomic groups than those from shallow samples. Fishes with northern distributions progressively decreased in their proportional representation with depth whereas those with widespread distributions increased.

**Conclusions/Significance:**

This study provides the first characterization of diversity patterns for bait-attracted fish species on continental slopes in New Zealand and is an imperative primary step towards development of explanatory and predictive ecological models, as well as being fundamental for the implementation of efficient management and conservation strategies for fishery resources.

## Introduction

Continental slopes extend from the outer edge of the continental shelf (∼100–200 m depth) to the base of the slope (∼4000 m) where abyssal plains begin. They are among the steepest environmental gradients on earth. Factors such as light, hydrostatic pressure, temperature, oxygen concentration, food availability, nature of water masses and substrate type all drastically change within a few hundred meters. The fauna are also known to change along this depth gradient. The fact that species composition changes with depth, resulting in distinct identifiable communities associated with the shelf, slope and abyssal plain has been virtually unchallenged since the 1970’s [1], but finer quantification and characterisation of such patterns for many parts of the world are still lacking. Diversity is increasingly acknowledged as essential for ecosystem functioning and the provision of goods and services to humanity [2], [3], [4], [5]. There has been relatively little work done, however, to understand the potential role of biodiversity for maintaining resilience and sustainability in marine ecosystems, compared to terrestrial or freshwater systems [6]. Elucidating patterns in bathymetric diversity is also important in order to complement our understanding of other known globally significant gradients of biodiversity, such as latitudinal or altitudinal gradients [7]. Understanding the mechanisms that control spatial variation in species richness may improve predictions of how biodiversity will respond to environmental changes and help the design of effective conservation strategies [8]. However, the causes underlying faunal turnover and diversity patterns on continental slopes have not been clearly identified [1]. A crucial first step is to accurately document how communities change along depth gradients.

**Figure 1:**
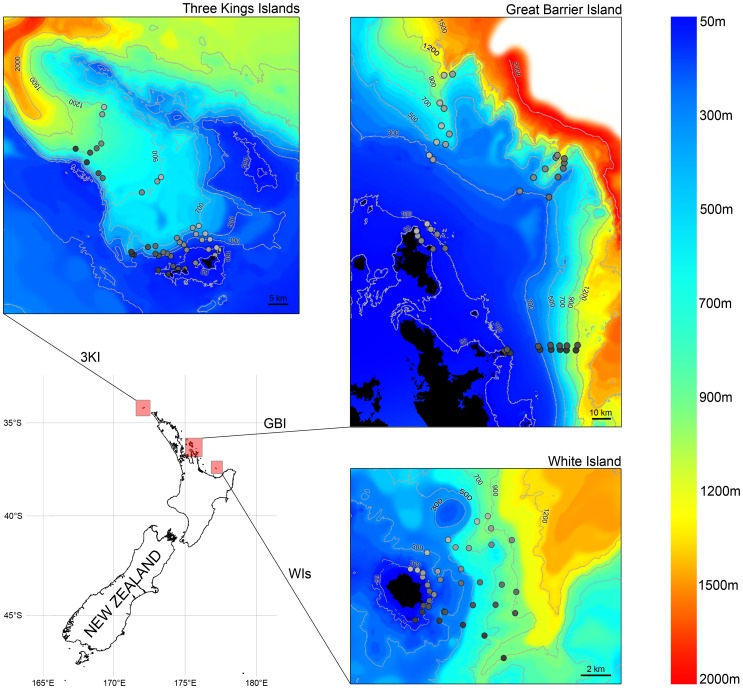
Map showing study area and stations sampled by baited remote underwater stereo-video (stereo-BRUVs). Three locations (White Island, Great Barrier Island and the Three Kings Islands) were sampled along six transects at each of 50, 100, 300, 500, 700, 900 and 1200 m depths. At White Island, samples at 1200 m were not taken due to logistical constrains. Bathymetry source: NIWA.

Fishes, which account for more than half of all living vertebrates [9], are an important component of slope and shelf communities, particularly through their feeding activity which can regulate trophic structure, and thus influence the stability and resilience of populations and food-web dynamics in aquatic ecosystems [10], [11]. Fisheries on continental margins also provide a significant amount of protein to the world’s human populations [12]. However, most fishes are highly mobile and therefore are difficult to sample, especially in topographically complex or deep habitats. Assessing the diversity of fishes on continental margins can be difficult because of the heterogeneity of potential habitats [13], variation in behaviour or abundances in the wild [14], and also because of the selectivity of particular fishing gear towards particular species [15], [16] or inevitable biases inherent in the use of remotely operated vehicles or submersibles [17], [18], which tend not to be deployed according to any structured sampling designs. In addition, sampling at and beyond the shelf break (often at depths beyond 200 m) is typically plagued with low levels of replication imposed by technical constraints [19], which significantly decreases the scope for potential inferences regarding patterns in diversity. In this context, baited remote underwater stereo-video system (stereo-BRUVs) deployments provide an extremely useful method to obtain standardized samples of fish communities which is non-destructive (unlike trawls) and can be easily replicated within a spatially structured sampling design for rigorous inferences [20].

It has been stated that the greatest diversity of marine species is found at some intermediate depth on the continental slope [21]. However, this is still debated, with numerous studies yielding inconsistent results for particular groups of organisms or locations [22]. Potential causal explanations for the observed patterns are also numerous, including the presence of oxygen minimum zones, changes in sediment grain size, topographic complexity, the degree of physical disturbance [23] or spatial and temporal variation in food availability [24], [25]. For example, Tolimieri [26] observed decreasing species richness of groundfish assemblages on the continental slope of the U.S. Pacific coast with increasing depth, reaching a minimum at about 600–900 m, followed by a slight increase. A similar pattern was observed for evenness [26]. In other studies, species richness of fishes on continental slopes either decreased continuously with depth [27], [28], [29], or showed a peak at some intermediate depth [30], [31]. The evenness of demersal fish assemblages was also found to decrease with depth down to 500 m in the northeast Atlantic [32].

Species diversity is often defined as the variety (richness) and relative abundance (dominance pattern or evenness) of species in a defined unit of study [33]. Relative abundances can also reflect the distribution of traits in a community, which in turn can indicate the strength and sign of intra-specific and inter-specific interactions [34]. The concept of diversity is, however, not limited to species richness and evenness. It can also include quantification of endemism [35], biogeographic distributions of species [36], information on the evolutionary history of the considered taxa (i.e., their phylogeny) or the functional behaviour or life-history characteristics of species [37]. For example, taxonomic distinctness quantifies the relatedness of species within a sample, based on the distance between species in a classification tree [38] and is used to evaluate the taxonomic diversity of a sample. The average taxonomic distinctness (AvTD) of a sample is defined as the average path-length between all pairs of species through a Linnean taxonomic tree. The variation in taxonomic distinctness (VarTD, [39]) is a measure of how variable the path-lengths are among species; it reflects the unevenness of the taxonomic tree for a given sample. There has been relatively little work on how taxonomic distinctness of fish assemblages changes with depth. On the continental slope of the U.S. Pacific coast, AvTD has been shown to be highest at approximately 500 m and lowest around 200 m, while VarTD slightly increased to 200–300 m depth, then sharply decreased at deeper depths [40]. This pattern was largely driven by high diversity of Chondrichthyes at 500 m compared to shallower or deeper strata. Off the coast of Corsica (Mediterranean Sea), the AvTD was higher on the continental shelf (60–120 m) than it was at deeper depths (250–570 m) where it was more stable [41], and the VarTD was lower on the upper slope (250–400 m) than on the continental shelf and lower slope (400–570 m). Zintzen et al. [42] showed no relationship with depth for AvTD, but found an increase in VarTD with depth in the Southwest pacific Basin (from 0 to ∼2000 m). This result indicated that clusters of species within specialized (unrelated) families occur at depth.

In this paper, we describe for the first time the fundamental changes in fish community structure and diversity along a depth gradient from 50 m to 1200 m, as obtained from replicated (*n* = 6) Baited Remote Underwater Stereo-Video (stereo-BRUVs) deployments within each of seven depth zones at each of three locations in north-eastern New Zealand. We hypothesized that: (1) changes in community structure would be very strongly affected by depth, and less affected by differences in locations; (2) diversity as richness would peak at some intermediate depth, where a variety of habitat conditions and thus fish adaptations to them overlap (3) evenness would increase with depth, as surface waters tend to have higher productivity that could lead to dominance in temperate waters; (4) average taxonomic distinctness would remain stable with depth as there is no expectation that groups of fishes with particular phylogenetic traits or histories would have colonized restricted zones of the continental slopes; (5) variation in taxonomic distinctness would increase with depth, showing diversification of species occurring only within specific groups; and (6) there would be decreased endemism at deeper depths, where potential connectivity is higher due to more uniform environmental conditions.

## Materials and Methods

### Location and Sampling Method

Stereo-video footage was collected between March 2009 and April 2010 at three locations, White Island (WI), Great Barrier Island (GBI) and the Three Kings Islands (TKI), in the north of New Zealand (Figure 1). At each location, video samples were taken along each of six replicate transects during daylight hours, and there were seven depth strata sampled per transect: 50, 100, 300, 500, 700, 900 and 1200 m, with the exception of WI where we could not sample the 1200 m depth stratum, due to logistical constraints. The resulting dataset comprised a total of 120 samples: 36 for WI (6 transects×6 depth strata), 42 for GBI (6 transects×7 depth strata) and 42 for TKI (6 transects×7 depth strata). Each deployment was located at least 500 metres away from any others in order to minimise the probability of a fish visiting the bait at one camera also being recorded by a neighbouring camera [43]. Each deployment was treated as an independent replicate and was deployed on the seabed for a minimum of two hours.

Seven separate stereo baited remote underwater stereo-video systems (stereo-BRUVs) were used for deployments, having a design adapted from the system used in shallow water by Harvey et al. [44] to work at greater depth. Two HD Sony handycams (models HDR-CX7 and HDR-CX500) were mounted on a stereo configuration, 0.7 m apart on a base bar inwardly converged at 8° to gain an optimised field of view. The bait consisted of two kilograms of *Sardinops sagax* (Jenyns 1842) that were thawed and chopped prior to distribution into two bait bags visible in the field of view. Lighting was provided by eight Royal Blue Cree XLamps XP-E LEDs, each delivering a radiant flux of 350–425 mW at wavelengths ranging from 450 to 465 nm (blue colour). This wavelength was chosen as a compromise between minimising fish repulsion, backscatter and reflection off the sides of silver-coloured fish whilst still illuminating a large enough field of view to be able to sample fish effectively [45]. With the blue LEDS, fish could be observed clearly within a distance of five metres from the camera system. Each unit was equipped with a sensor (Star Oddi DST or centi TD) that measured depth to a precision of 0.4–0.6% of the depth range (e.g., ±1 m at 50 m depth and ±9 m at 1200 m depth). The time required for camera systems to fall to required depths was relatively short, ranging from less than one minute at 50 m to an average of 12 minutes at 1200 m depth, so bait was still fresh at landing.

### Image Analysis

The Sony CX7 and CX500 camcorders recorded video imagery in MPEG Transport Stream format (MTS) which was converted to high-definition MPEG format [46]. At the beginning and completion of the field work, the stereo-BRUVs were calibrated following procedures outlined in Harvey and Shortis [47] using Cal software (v1.32, www.seagis.com.au). With the stereo capability, sizes of fishes can also be measured and analyzed, but for present purposes, we focused on identification and counts of numbers of individual fish species only. We used the software EventMeasure (www.seagis.com.au) to annotate the time that we first saw a species of fish on the video and to keep tally of the maximum number of individuals that we saw of any one species at any one time. Due to the concern that individual fish may be counted repeatedly upon leaving and re-entering the field of view over the two-hour period of sampling, the maximum number of individuals of the same species appearing at the same time (MaxN, [48]) was used as a conservative estimate of the number of fish seen of any one species from each stereo-BRUVs deployment [20], [49], [50]. The horizontal orientation of the stereo-BRUVs usually provided clear lateral shots of fishes, which greatly increased the accuracy of their identification. Fish identification was carried out using published [51], [52] and unpublished Museum guides to New Zealand fishes, and by taxonomic specialists for difficult groups like Macrouridae, Somniosidae, Centrophoridae and Squalidae. From recent unpublished work, it appears that the hagfish *Eptatretus cirrhatus* may actually be a complex of two species with very similar morphological characteristics. Consequently, we refer here to this taxon as *Eptatretus cf.cirrhatus*. Some individuals could be identified to the species level but have not been formally described yet. These species were named using classical binomial Operational Taxonomic Units (OTUs), namely as *Genus* sp.1, *Genus* sp.2 or *Genus* n.sp.

Each species recorded from the three locations sampled was assigned as endemic or not and to one of three geographic distributions within the New Zealand region (northern, southern or widespread), based on published records (e.g. [53], [54], [55]) and unpublished data (Te Papa database). Definitions are (1) endemic: unique to an area within the New Zealand region, (2) northern: known mostly from an area north of Cook Strait, (3) southern: known mostly from an area south of Cook Strait and (4) widespread: ranging from North Cape in the north to Stewart Island in the south. Some taxa had unknown distributions because identification was only possible to genus level and some genera had more than one species and more than one type of distribution. Counts of endemic species and of species having different biogeographic distributions were also summarised by depth strata and the patterns described.

### Statistical Analyses

The calculation of all diversity measures and all multivariate analyses of community structure were done using the PRIMER v6 computer program [56] with the PERMANOVA+ add-on package [57].

#### Univariate indices of diversity

Several univariate indices of diversity were calculated and examined for their potential relationship with depth. These included: species richness (the number of species per sample unit), Simpson’s measure of evenness [33], [58], [59], average taxonomic distinctness (Δ^+^) [38] and variation in taxonomic distinctness (Λ^+^) [39].

Simpson’s (1949) measure of dominance within a sample unit, directly interpretable as the probability that any two individuals chosen randomly from the same sample belong to the same species, can be defined as:
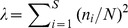
where *S* is the number of species, *n_i_* is the number of individuals in the *i*th species and *N* is the total number of individuals. A corresponding measure of evenness or equitability, which is also standardised for the number of species in the sample [33], [59], is defined as *E* = (1/*λ*)/*S*, which was the measure of evenness examined here.

Although classical measures of diversity focus on species-level units, an additional concept of diversity is obtained by considering the taxonomic relationships among species. The average taxonomic distinctness of a sample (AvTD or Δ^+^, [38]) is defined as the average path length between all pairs of species through a Linnean taxonomic tree, which here included the levels of species, genus, family, order and class. The largest path length (i.e., between species in different classes) is set to 100 and the steps between different hierarchical levels of the tree (i.e., from species to genus, genus to family, etc.) were set to be equal (although the original description of the measure allows for variation in the weights associated with these different steps, see [38] for further details). A large value of taxonomic distinctness indicates broad taxonomic diversity in the sample. A sample having only multiple species within a single genus will have lower taxonomic distinctness than a sample having multiple species from different families. Another very important attribute of this measure that distinguishes it from most other univariate measures of diversity is that it is not dependent on sample size [38].

Fishes are a relatively cohesive and taxonomically well-studied group, so the use of the Linnean classification as a proxy for phylogenies is reasonable here. The taxonomic tree was produced using the latest taxonomic information available, based on Nelson [60] and on the Catalog of Fishes from the California Academy of Sciences [61].

A further index of taxonomic biodiversity known as the variation in taxonomic distinctness (VarTD or Λ^+^, [39]) is a measure of how variable the path-lengths are among species; it reflects the unevenness of the taxonomic tree for a given sample. Thus, Λ^+^ would be relatively large for a sample that contained clusters of species belonging to the same genus (contributing short path-lengths), but where the different clusters themselves are not necessarily closely related (contributing long path-lengths). Importantly, the variation in taxonomic distinctness (Λ^+^) is independent of the average taxonomic distinctness (Δ^+^), so measures a different aspect of the community’s taxonomic structure, and, like Δ^+^, Λ^+^ is also independent of sample size.

**Figure 2:**
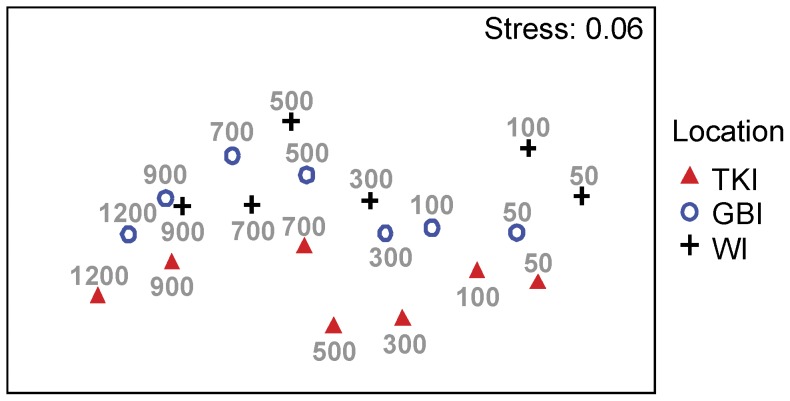
Ordination of fish assemblages with depth at each of three New Zealand locations. Non-metric MDS plot on the basis of Jaccard resemblances between fish assemblages consisting of averages from *n* = 6 baited remote underwater stereo-video system deployments within each combination of depth (50, 100, 300, 500, 700, 900 or 1200 m) and location (White Island, Great Barrier Island or the Three Kings Islands).

#### Multivariate analyses

Variation in the structure of fish assemblages along the depth gradient was examined on the basis of two different resemblance measures: Jaccard dissimilarity [62] and taxonomic dissimilarity (Γ^+^, [63]). The Jaccard measure is calculated from presence/absence data and is directly interpretable as the proportion of unshared species, excluding joint-absences, as follows:
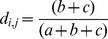
where *a* is the number of species that samples *i* and *j* have in common, *b* is the number of species in sample *i* not shared with sample *j* and *c* is the number of species in sample *j* not shared with sample *i*.

Taxonomic dissimilarity (Г+) is a natural extension of the measure of average taxonomic distinctness [63]. It is defined as
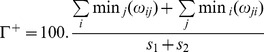
where *ω_ij_* is the path length between species *i* and *j* and *s*_1_ and *s*_2_ are the numbers of observed species in samples 1 and 2, respectively. Г^+^ is the mean of all path lengths through a tree (in our case, a standard Linnean taxonomic tree) between each species in one sample and its closest relation in the other sample. This dissimilarity measure is particularly useful for comparing samples having zero or few species in common. As the entire taxonomic structure is used to compute Г+, samples with no species in common can have dissimilarities <100 if they share some branches of the taxonomic structure (e.g., if some of their species belong to the same family).

**Figure 3:**
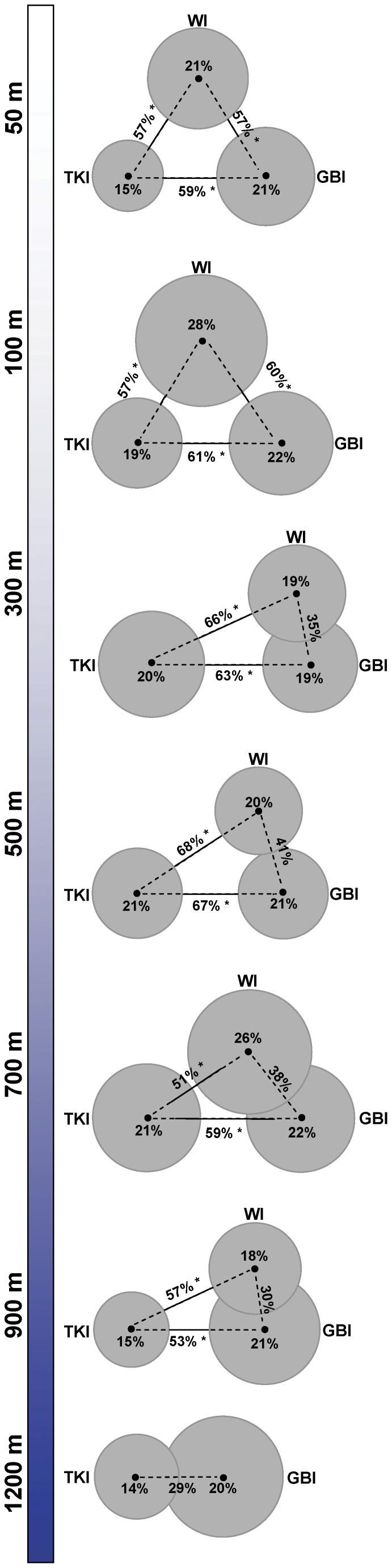
Pair-wise comparisons of fish assemblages among locations at each depth. Graphical representation of PERMANOVA pair-wise tests comparing fish assemblages among locations separately for each depth stratum on the basis of the Jaccard resemblance measure. Triangle side lengths are proportional to the Jaccard dissimilarities between location centroids. Circle radii are proportional to a pseudo multivariate standard error around the group centroid for each location within each depth. Due to the large number of comparisons, a conservative significance level of α = 0.01 was used. Significant differences (*p*<0.01) are marked by an asterisk (*). Partitioning was done using Type I (sequential) sums of squares and *P*-values were obtained for each term using 9999 permutations under the reduced model. Similar results were obtained using the taxonomic resemblance measure Г^+^.

In order to visualize the patterns of differences in community structure among locations along the depth gradient, an ordination was done using non-metric multidimensional scaling (nMDS) of Jaccard (and also taxonomic) dissimilarities, calculated on the depth-by-location averages from *n* = 6 samples. The goodness-of-fit of the resulting two-dimensional nMDS plot was measured using Kruskal’s stress formula I [64]. In order to visualise relationships among species, a separate nMDS plot was produced from Jaccard resemblances calculated among species. Species appearing frequently together in video samples will have symbols on this nMDS plot that are relatively close together. To focus on the most abundant/common species in the dataset, only the 40 most important species (namely, those which, for every sample, accounted for >5–6% of the total abundance of the sample) were included in this nMDS plot among species.

The statistical significance and relative importance of the factors of location (Lo, a fixed factor with 3 levels), depth stratum (De, a fixed factor with 7 levels), transects (Tr, a random factor nested within Lo) and the interaction LoxDe were investigated using permutational multivariate analysis of variance (PERMANOVA, [65]) on the full set of data at the replicate level. Although the design was unbalanced (with one missing cell – the deepest stratum at WI), correct multivariate analogues to traditional expectations of mean squares can be used to construct pseudo-*F* ratios [66] and obtain appropriate tests by permutation (e.g. [67]) for each term in the model, using the software PERMANOVA+ [57]. These analyses were done using type I (sequential) sums of squares and *P*-values were obtained using 9999 permutations under a reduced model [68]. Results were not affected in any way by changing the order of terms in the sequential analysis.

After discovering a significant depth-by-location interaction (see results), pair-wise tests were done among locations separately within each depth stratum. Results were graphically displayed by showing, for each depth, a triangle where the Euclidean distances among the three locations (as three points) are directly proportional to the Jaccard dissimilarity between each corresponding pair of centroids. The variability in species identities within each depth-by-location cell (i.e., the within-group dispersion) relevant for comparing the centroids was then superimposed on each point as a circle, with radius equal to a pseudo multivariate standard error (*SE*), measured in Jaccard units. This *SE* was estimated by (1) calculating the individual deviations (in Jaccard space) of each sample from its depth-by-location centroid (obtained using the function PERMDISP [69] in the PERMANOVA+ add-on package [66]), (2) calculating the multivariate variation (*V*) by summing the squares of these deviations and dividing by (*n* –1), where *n* = 6 is the number of replicate deployments for each depth stratum within each location and (3) calculating *SE* = (*V*/*n*)^1/2^.

**Table 1:** PERMANOVA results for the analysis of fish assemblages on the basis of the Jaccard resemblance measure in response to location, depth stratum and transect.

Source	df	MS	Pseudo-*F*	*P*	Sqrt(Componentof variation)	%
Lo	2	15,898	6.38	0.0001	18.505	6.8%
De	6	27,086	11.57	0.0001	38.486	29.4%
Tr (Lo)	15	2,491	1.06	0.1413	4.780	0.5%
Lo×De	11	7,300	3.12	0.0001	29.079	16.8%
Res	83	2,341			48.388	46.5%
Total	117					

Location (Lo, fixed, 3 levels), depth stratum (De, fixed, 7 levels) and transect (Tr, random, nested within Lo, 6 replicates). Similar results were obtained using the taxonomic resemblance measure (Γ^+^). Partitioning was done using Type I (sequential) sums of squares and *P*-values were obtained for each term using 9999 permutations under the reduced model. The estimated sizes of effects for each term in the model are shown in Jaccard units as the square root of the components of variation, and variation attributable to each individual term is also expressed as a percentage of the total (%). Note that 2 of the original 120 samples were omitted prior to analysis, as there were no fish recorded in those deployments.

### Ethic Statement

Sampling was made in waters of the New Zealand Exclusive Economic Zone under a New Zealand Ministry of Fisheries Special Permit (#444), pursuant to Section 97(1)(a)(i) and (ii) of the Fisheries Act 1996. Client number for this permit: 9360085. This study strictly involved video sampling and no animals or living organisms were collected.

## Results

Video S1 shows some representative examples of video images for a range of species and habitats sampled at each of the three locations and within each depth stratum. From the 120 video samples, a total of 2,674 specimens in 137 fish taxa were identified (see Tables S1, S2, S3 for a complete list of species). Of these taxa, 106 were confidently identified to species level, while 19 and 12 taxa were only identified to the level of genus and family, respectively. In terms of taxonomic diversity, the specimens identified belonged to 98 genera in 66 families and 19 orders. The number of taxa per sample varied from 0 (2 samples at 100 m in WI had no fish) to 19, with median richness per depth stratum ranging from 5 (at 300 m) to 10.5 (at 50 m). The habitat types sampled were diverse, ranging from mud-covered bottoms to reef-like structures (see Figure S1). Although most of the different types of habitat were sampled at each depth stratum, reefs were sampled from depths of 50–700 m only and muddy habitats were only found at depths greater than 500 m.

The Spearman rank correlation between the Jaccard and taxonomic dissimilarity matrices was extremely high (*ρ* = 0.97), so, for simplicity, results are presented using only the classical Jaccard measure. Gradual changes in the structure of fish assemblages with increases in depth were very clear in terms of species shared (as measured by the Jaccard measure, Figure 2) and also in terms of taxonomic resemblance. While the turnover in species identities with depth was clearly visible along the X-axis of the Jaccard nMDS plot, differences among the three locations were evident along the Y-axis. The samples from TKI (near the bottom of the plot) were clearly separated from WI and GBI which were more similar at any particular depth stratum. This was particularly evident at 500 m, where a large disjuncture between TKI and the other locations was observed. Samples taken at 700 m, 900 m and 1200 m depth were more clustered together, indicating greater faunal similarity occurs at depth across the region as a whole.

**Table 2:** PERMANOVA pair-wise comparisons of fish assemblages at different depths done separately at each location on the basis of the Jaccard resemblance measure.

Pair of depth strata being compared (m)	*P* value		
	TKI	GBI	WI
50 *vs*100	0.047	0.103	0.106
50 *vs* 300	**0.003**	**0.002**	**0.002**
50 *vs* 500	**0.002**	**0.003**	**0.002**
50 *vs* 700	**0.002**	**0.003**	**0.008**
50 *vs* 900	**0.003**	**0.004**	**0.002**
50 *vs* 1200	**0.002**	**0.003**	–
100 *vs* 300	0.012	0.019	0.036
100 *vs* 500	**0.003**	**0.008**	0.020
100 *vs* 700	**0.004**	**0.005**	0.043
100 *vs* 900	**0.003**	**0.004**	0.013
100 *vs* 1200	**0.002**	**0.004**	–
300 *vs* 500	0.135	0.016	0.014
300 *vs* 700	0.101	**0.005**	0**.**017
300 *vs* 900	**0.003**	**0.002**	**0.003**
300 *vs* 1200	**0.002**	**0.002**	–
500 *vs* 700	0.096	0.020	0.048
500 *vs* 900	**0.002**	**0.004**	**0.002**
500 *vs* 1200	**0.003**	**0.002**	–
700 *vs* 900	**0.002**	0.043	0.033
700 *vs* 1200	**0.002**	**0.003**	–
900 *vs* 1200	**0.007**	0.022	–

Partitioning was done using Type I (sequential) sums of squares and *P*-values were obtained for each term using 9999 permutations under the reduced model. Similar results were obtained using taxonomic resemblances Γ^+^. Due to the large number of comparisons, a conservative level of α = 0.01 was chosen. Significant *p*-values (<0.01) are shown in bold.

There was a significant interaction between location and depth (Table 1), indicating that the degree of difference among locations in the structure of fish assemblages depends on depth and also that depth effects depended on the location. The PERMANOVA partitioning and the ordination of samples in the nMDS plot showed that this interaction was small in size, however, compared to the main effect of depth (Figure 2, Table 1). Depth as a main effect explained nearly 30% of the variability in the data, while locations explained only about 6.8% and the interaction accounted for 16.8% (Table 1). Pair-wise comparisons among locations separately within each depth stratum provided more insights into the origin of this interaction (Figure 3). The fish assemblages at TKI were significantly different from those at WI or GBI at every depth, with the exception of the 1200 m depth stratum where the fish assemblages at TKI did not differ significantly from those at GBI. In shallow water (50 m and 100 m), the fish assemblages were significantly different between WI and GBI, but there were no significant differences between these two locations for any of the deeper strata (300 m and deeper).

**Figure 4:**
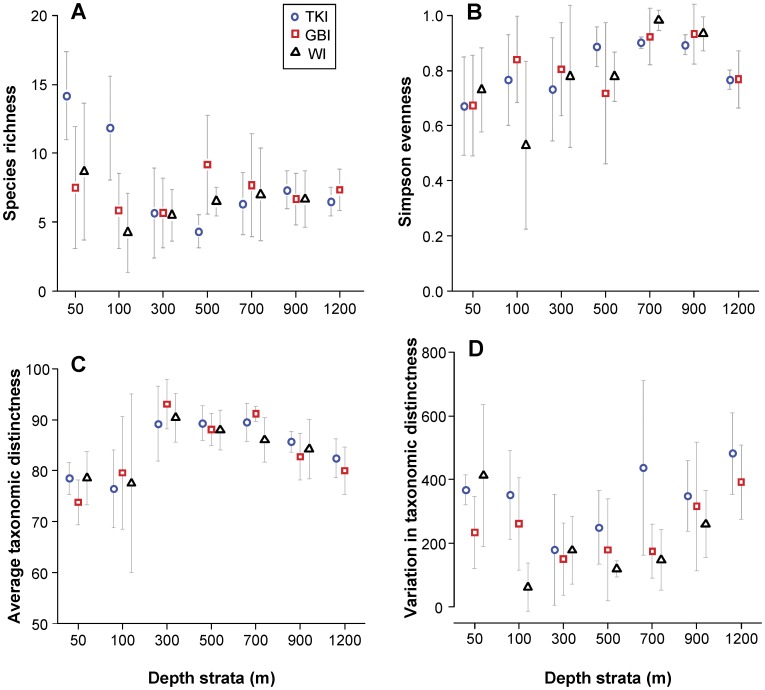
Relationships between fish biodiversity indices and depth. Plots showing the mean ±1 SD (*n* = 6) for each of (a) species richness, (b) Simpson’s evenness, (c) average taxonomic distinctness, Δ^+^ and (d) variation in taxonomic distinctness, Λ^+^ for fish assemblages *versus* depth at each of three locations.

Furthermore, at each location, most fish assemblages were significantly different between all pairs of depth strata (Table 2). However, there were no significant differences detected between 50 and 100 m depth strata at any of the locations. Similarly, comparisons between adjacent depth strata at intermediate depths (100 *vs* 300 m, 300 *vs* 500 m, and 500 *vs* 700 m) were not statistically significant at any location, indicating a gradual change in faunal composition as seen in the nMDS plot (Figure 2). This was also true at deeper depths for WI and GBI, but at TKI there was apparently more stratification, as significant differences were found between adjacent pairs of assemblages for the deep strata (i.e., 700 *vs* 900 m and 900 *vs* 1200 m, Table 2).

**Figure 5:**
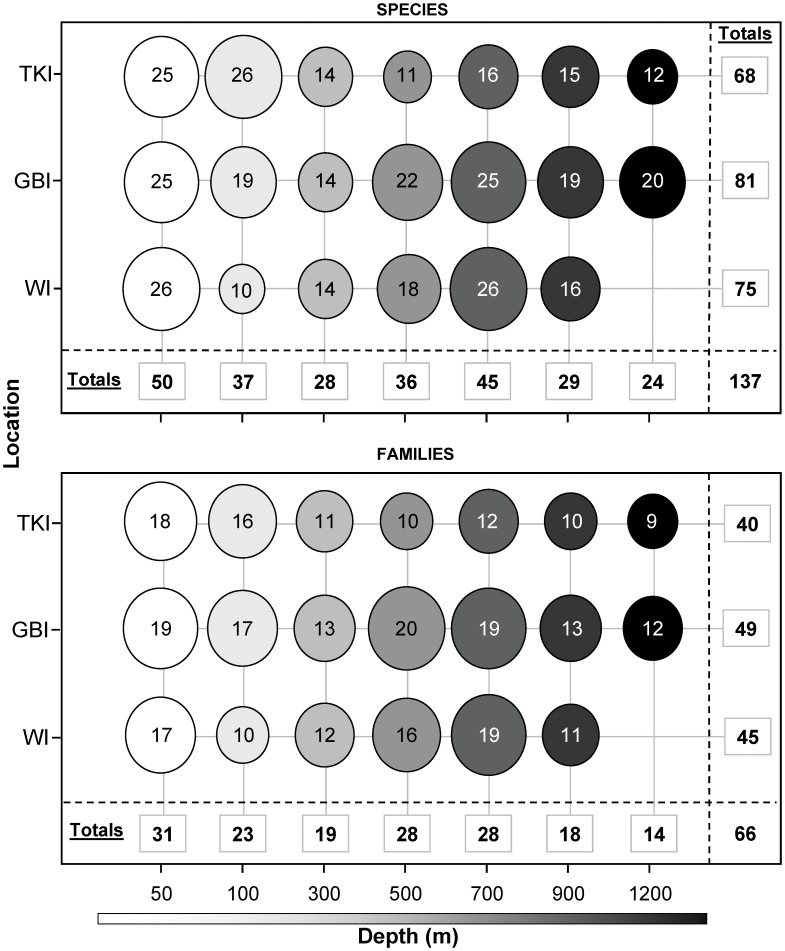
Family and species richness of fishes identified from video samples by depth and location. Each circle pools the information obtained from *n* = 6 two-hour video deployments and its diameter is proportional in size to the richness value at one location. Overall totals for depths and locations are given in the marginal columns and rows, respectively, with the grand total given in the bottom right-hand corner.

Patterns in univariate diversity measures along the depth gradient were examined separately at each of the three locations (Figure 4). Average values of species richness ranged from 4.3 to 14.2, Simpson’s evenness from 0.53 to 0.98, average taxonomic distinctness (△^+^) from 74 to 93 and variation in taxonomic distinctness (Λ^+^) from 62 to 482. Species richness showed a clear peak in shallow depth strata with particularly higher average richness observed at the northern location (TKI), followed by a decrease beyond 100 m and a stable common trend of an average number of ca. 7 species recorded per video deployment from 700 to 1200 m (Figure 4a). The decrease in species richness from 100 to 300 m was particularly sharp for TKI. Minima in average species richness occurred deeper at the northern sites, with TKI, GBI and WI having their minima at 500, 300 and 100 m, respectively. It is also clear that in shallower strata, the variability among samples in species richness was greater than in deeper strata. From 700 m onwards, variability in species richness for all locations decreased with depth. Evenness was also more variable at shallow depths, but with a trend of increasing evenness to a depth of about 700 m, followed by a decrease in mean evenness to 1200 m (Figure 4b). Similarly, average taxonomic distinctness △^+^ was also unimodal, but with a peak at 300 m for all locations (Figure 4c). The variability of △^+^ was especially high among samples at the 100 m depth stratum and low at deeper depths. Finally, the variation in taxonomic distinctness Λ^+^ first decreased sharply from 50 to 300 m, then increased beyond 500 m depth, indicating that the taxonomic tree of deeper samples had more variable path-lengths than did shallower samples (Figure 4d). Species from deep samples belonged to more distant taxonomic groups that those from shallow samples.

**Figure 6:**
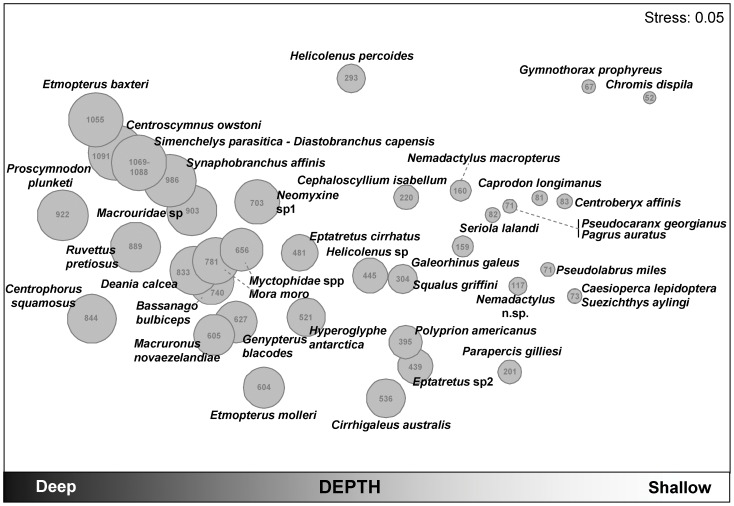
Ordination of fish species associations, with average depth as bubbles. Non-metric MDS plot of the Jaccard resemblances among the 40 most important fish species in the dataset, based on their frequencies of occurrences. Species which co-occur together often will have high Jaccard similarity, and will be placed by the nMDS algorithm relatively close together on the plot. The 40 most important species are those which, for every sample, account for >5–6% of a sample’s total abundance. The relative average depth of the samples at which each species occurred is shown using scaled bubbles. The average depth (in metres) at which each species was recorded is also shown numerically in the centre of each bubble.

Pooling the *n* = 6 samples taken at a given depth and location, species richness ranged from 10 to 26 (Figure 5). At each location, lower total species richness was displayed at some intermediate depth, with relatively high species richness in the shallow (50 m) and deeper strata (500–700 m). This low total species richness was observed at 100, 300 and 500 m for WI, GBI and TKI, respectively. A similar trend was observed at the family level. Compared to the species level, the richness at family level tended to more sharply decrease at the deepest depths, indicating clusters of species within fewer families.

**Figure 7:**
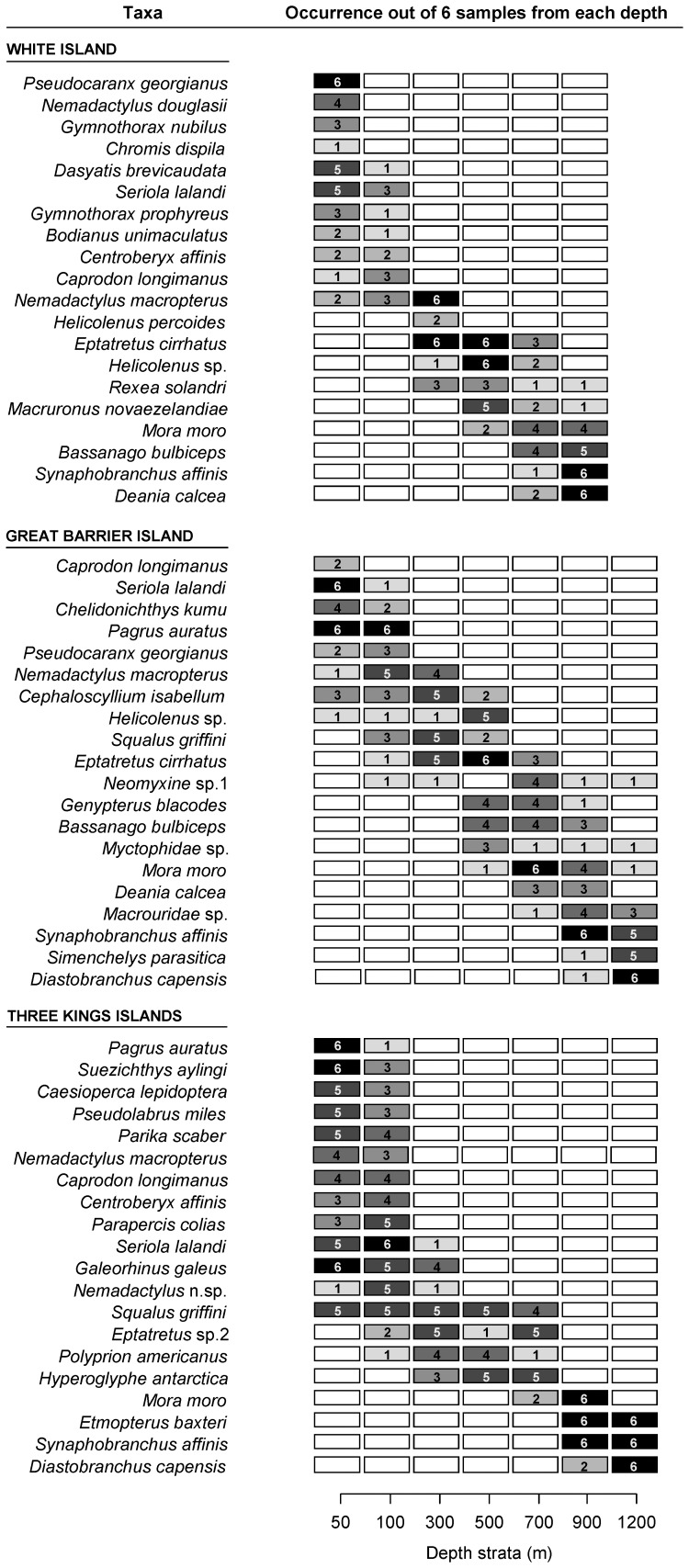
Occurrence of the most abundant fish taxa with depth. The frequencies of occurrence (out of n = 6 samples) for each of the 20 most abundant taxa at each depth and across each of the three locations are presented. For each location, the species are ordered from shallow to deep, with the most common species at a particular depth being cited first.

Associations between species are shown in Figure 6, and the occurrences in the different depth strata of the 20 most abundant taxa at each location are shown graphically in Figure 7. Typically, a set of species associated with one another (Figure 6) occurred primarily within certain depth strata (Figures 6 and 7). A depth gradient is also evident in these inter-specific associations (Figure 6), as illustrated by examining the average depth of occurrence for each species. In shallow depth strata, the most abundant species were similar across all locations and the fish fauna was characterized by greater frequencies (and co-occurrence) of *Pseudocaranx georgianus* (Cuvier 1833), *Pagrus auratus* (Forster 1801), *Centroberyx affinis* (Günther 1859), *Caprodon longimanus* (Günther 1859) and *Seriola lalandi* Valenciennes 1833 (Figure 7). At TKI, *Caesioperca lepidoptera* (Forster 1801), *Suezichthys aylingi* Russell 1985, *Pseudolabrus miles* (Schneider & Forster 1801) and *Nemadactylus* n.sp were also closely associated at typically 70–120 m depth. TKI had the greatest range in fish abundances, as measured using sum of the values of *MaxN* pooled across the *n* = 6 samples (i.e., *N* = 1–367), with three species showing greatest total abundances: schooling *Caprodon longimanus* (*N* = 367) and *Caesioperca lepidoptera* (*N* = 197) and *Suezichthys aylingi* (*N* = 192). *Nemadactylus macropterus* (Forster 1801), *Galeorhinus galeus* (Linnaeus 1758), *Cephaloscyllium isabellum* (Bonnaterre 1788), *Parapercis gilliesi* (Hutton 1879) and *Squalus griffini* Phillipps 1931 were typical of the 150–300 m depth range. *Polyprion americanus* (Bloch & Schneider 1801), an undescribed species of hagfish *Eptatretus* sp.2 and *Cirrhigaleus australis* White, Last & Stevens 2007 were closely associated at average depths of 390 to 540 m, especially at TKI. *Macruronus novaezelandiae* (Hector 1871) and *Genypterus blacodes* (Forster 1801) were observed in samples having an average depth of just over 600 m. In the depth band 650 to 830 m, *Deania calcea* (Lowe 1839), *Bassanago bulbiceps* Whitley 1948, *Mora moro* (Risso 1810) and Myctophidae spp commonly co-occurred. The deepest station had three synaphobranchids (*Simenchelys parasitica* Gill 1879, *Synaphobranchus affinis* Günther 1877 and *Diastobranchus capensis* Barnard 1923) and two sharks (*Centroscymnus owstoni* Garman 1906 and *Etmopterus baxteri* Garrick 1957) as characteristic species. Generally, the most abundant species occurred within relatively narrow depth bands (Figure 7). A few species, like the hagfishes *Neomyxine* sp.1 and *Eptatretus cf.cirrhatus* (Forster 1801) or the dogfish *Squalus griffini* Phillipps 1931 occurred across a depth range of more than 700 m.

**Table 3:** Occurrence by depth strata of fish species having different biogeographical distributions† in New Zealand waters.

Depth (m)	Distribution						Total	
	Northern		Southern		Widespread			
	All	Endemic	All	Endemic	All	Endemic	All	Endemic
50–99	22 (33%)	4 (22%)	0	0	20 (17%)	8 (36%)	42 (23%)	12 (29%)
100–299	20 (30%)	3 (17%)	1	1	17 (15%)	4 (18%)	38 (21%)	8 (20%)
300–499	10 (15%)	5 (28%)	0	0	8 (7%)	1 (5%)	18 (10%)	6 (15%)
500–699	8 (12%)	1 (6%)	0	0	12 (10%)	2 (9%)	20 (11%)	3 (7%)
700–899	5 (7%)	3 (17%)	0	0	21 (18%)	0 (0%)	26 (14%)	3 (7%)
900–1200	2 (3%)	2 (11%)	0	0	39 (33%)	7 (32%)	41 (22%)	9 (22%)
**Total**	67	18	1	1	117	22	185	41

† Definitions of northern, southern or widespread biogeographical distributions are given in the text. Results are also presented for species known to be endemic to New Zealand waters.

Some species were common at all three locations: *Seriola lalandi* (*N* = 34, 27, 15, for TKI, GBI and WIs, respectively), *Mora moro* (*N* = 20, 17, 12), *Helicolenus* sp. (*N* = 14, 14, 19), *Deania calcea* (*N* = 3, 8, 9), *Gadomus aoteanus* (*N = *3, 4, 2), *Genypterus blacodes* (*N* = 3, 3, 3). However, most species were common or abundant at just one location and absent or present in low numbers elsewhere. For example, at TKI, prevalent taxa included the hagfish *Eptaptretus* sp.2 (*N* = 33, 0, 0), *Parika scaber* (*N* = 30, 1, 1), *Nemadactylus* n.sp. (*N* = 29, 1, 1), *Polyprion americanus* (*N* = 18, 0, 1) and *Proscymnodon plunketi* (*N* = 15, 1, 0). Conversely, some species that were absent or had low abundance at TKI were more numerous at the other locations: e.g., *Eptaptretus cf.cirrhatus* (*N* = 0, 53, 57), *Pseudocaranx georgeanus* (*N* = 7, 14, 29), *Bassanago bulbiceps* (*N* = 3, 16, 10), *Cephaloscylium isabellum* (*N* = 3, 17, 4) and *Rexea solandri* (*N* = 1, 8, 8). *Nemadactylus macropterus* was common at TKI, but nearly twice as abundant at GBI and four times as abundant at WI.

Fishes endemic to New Zealand occurred at all three locations (TKI, GBI, WI) and within most depth strata sampled, ranging from a minimum of 48 m to a maximum of 1275 m depth (Table 3 and Tables S1, S2, S3). The percentage of endemic fishes was highest at TKI (22.1%) and GBI (21.0%), and lowest off WI (12.0%). Interestingly, the percentage of endemic fishes was highest at shallow depths (50 m or 100 m) and also at very deep depths (900 m or 1200 m), where they made up>20% of the fish taxa that were recorded (Table 3). The occurrence of endemics was lowest, however, at intermediate depths (from 300–800 m).

In terms of geographical distributions, there was only one species recorded from the video footage that could be classified as a southern species – the girdled wrasse (*Notolabrus cinctus*), although it has been recorded at TKI before [51]. It was found at just one location and depth stratum (TKI, 109 m). The percentage of fishes with a particular biogeographical distribution varied with depth. Widespread species were least common at middle depths (300–700 m, 17.1%) and most common at shallow depths (50–300 m, 31.6%) and deeper depths (700–1200 m, 51.2%). Northern species were most common in shallow water (50–300 m, 62.7%), progressively decreasing in numbers with depth (700–1200 m, 10.5%).

## Discussion

There was a strong gradient of change in the diversity and community structure of fishes observed using stereo-BRUV deployments along the New Zealand continental shelf and slope. Clear turnover in the identities of species was observed with increasing depth, and this was mirrored by turnover in taxonomic resemblances. Although video deployments at different depths were separated spatially by just hundreds of metres, depth effects greatly exceeded location effects, which spanned hundreds of kilometres, and regional similarities in the fish fauna became apparent at the deeper depths, where differences among locations were no longer statistically significant. Traditionally, fish assemblages have been divided into shelf (50 to ∼300 m), upper slope (300 to ∼900 m), middle slope and lower slope (2000 to ∼4000 m) assemblages [70]. This pattern has been confirmed at different locations [27], [71], [72], [73]. Our observations suggest a more gradual continuum of change in fish assemblages with depth. Although an initial zone does appear to exist up to ∼100 m, comparisons of samples taken from deeper adjacent depth strata (i.e., 100 *vs* 300 m, 300 *vs* 500 m, and 500 *vs* 700 m) were not strongly dissimilar, indicating a progressive turnover of species rather than a distinct zonation of species with restricted depth ranges. At 700 m depth, another pattern emerged. The fish fauna of deeper strata (700 to 1200 m) displayed faster turnover, however, especially at the Three Kings Islands, the most northern location. This depth range (700–1200 m) may represent a zone of transition between the upper and lower-slope fauna. In his extensive data analysis of New Zealand demersal fish fauna, Francis [73] identified inshore (0–60 m), shelf (50–300 m), upper slope (400–740 m) and mid-slope (720–1320 m) assemblages. Similar boundaries and affinities in the composition of mid-slope fauna have been documented in south-eastern and western Australia [74], [75]. Comparing the species lists from our study and Koslow [74], we find that more than 50% of the New Zealand species are found on both sides of the Tasman Basin for the depth range from 700 to 1200 m. Antarctic Intermediate Waters of low salinity extend to New Zealand within this particular depth range, and might be one of the drivers of such a distinct zone [76], coinciding with a distribution of relatively similar fish fauna across the South Pacific and Atlantic [74], [75], [77], [78].

Both individual sample and location-scale species richness were relatively high in the shallower strata (50 m) and then decreased to reach a minimum at a depth which depended on the location. Minimal species richness at intermediate depths (e.g., at 500 m on the U.S. Pacific coast, [26]) has been linked with an oxygen minimum zone [79]. In our case, however, the shallow depth at WI where minimal richness occurred (100 m) is more likely to have been caused by toxic volcanic activity at that particular location. In southern New Zealand waters, Some possible explanations for an increase in the depth at which minimum richness occurs at northerly locations include: (i) greater stability in seasonal climate conditions, (ii) greater substratum heterogeneity in deeper waters at TKI, or (iii) two different oceans meeting at TKI to generate oceanographic confluence, mixing and upwelling, thereby increasing overall richness and also extending the depth at which greater richness occurs.

Overall, evenness tended to increase with depth, in agreement with our prediction that fish species in deeper habitats would be more uniformly abundant due to a reduced input of energy. A drop in evenness was observed for the 1200 m strata. These deep samples were dominated by two species of synaphobranchid eels (*Diastobranchus capensis* and *Synaphobranchus affinis*) which appeared in relatively high numbers compared to other species. At White Island, low average evenness occurred at 100 m, explained by having two samples with no species and the other samples dominated by either *Centroberyx affinis* or *Nemadactylus macropterus*. These species form schools, especially in deeper waters [51]. On the U.S. Pacific coast, evenness in groundfish assemblages was observed to be high in shallow water (200–500 m), lower at 600–900 m and then higher again at 1000–1200 m [26]. It is difficult to discern spatial or environmental patterns of evenness from trawl data such as these, however, due to the selectivity of trawling gear for particular types of species [26].

Average taxonomic distinctness (highest at 300 m) showed an opposite trend to the variation in taxonomic distinctness (lowest at 300 m). The low value of average taxonomic distinctness at shallow depths can be explained by the predominance of species which are closely associated in the taxonomic tree. Between 300 and 700 m, the proportion of Chondrichthyes relative to Osteichthyes was larger than at either shallower or deeper depths, leading to higher AvTD values. Both ends of the depth spectrum measured here showed high variation in taxonomic distinctness, indicating taxonomically distinct clusters of highly related species occur at these two extremes (i.e. 50 m and 1200 m) [42]. It appears that some lineages have specialized for the deep-sea environment [80]; members of the Macrouridae and Alepocephalidae have been noted to dominate in deep-sea environments [16], along with Synaphobranchidae, Moridae and Ophiididae [81].

In addition to rapid faunal turnover beyond 700 m, we observed increasing similarities among the fauna of the deeper samples across locations. A decrease in fish diversity with depth may reflect a decrease in habitat heterogeneity [82]. Turnover could also be driven at large scales by organic carbon flux to the seafloor [83], as would be predicted by a general positive species-energy relationship [8]. Rex and Etter [83] suggested that limited food supply at depth for larger animals would also limit population size, which in turn would negatively affect diversity [8]. The extent to which there are increased affinities in fish assemblages with increasing depth, despite large separations among locations, as found here, may or may not be maintained at larger spatial scales. For example, deep-sea demersal fish assemblages of seamounts have strong affinities at the regional scale (100 to a few 1000’s km), but not at larger oceanic scales, where other factors, probably historical, came into play [77].

In this study, we identified 137 taxa (mostly to species) from 120 stereo-BRUV deployments. The number of taxa observed here by stereo-BRUVs compares favourably with the diversity measured in other studies using trawls, although we used a less intensive sampling scheme. For example, in New Zealand’s subantarctic region, Jacob [84] sampled 102 fish taxa from 363 bottom trawls at depths ranging from 80 to 787 m.

Our study recorded a total of 117 species of fishes with widespread distributions and 67 species of northern fishes from the three areas sampled. This is the first time that widespread and northern fishes have been recorded occurring in such high numbers and extending to such great depths (1275 m) off New Zealand. However, fishes with northern distributions progressively decreased in their proportional representation with depth whereas those with widespread distributions increased. One explanation for this could be the tolerance of species to water temperature. It is perhaps surprising, however, that northern species occur at all past 300 m depth, where temperatures are usually below 10°C. Here, we documented northern species occurring in all depth strata sampled down to 1275 m, where bottom temperatures of 4–6°C are the norm. Surface temperatures this low would be found well south of subantarctic New Zealand in the Southern Ocean. Thus, the dynamics of individual species’ distributions with depth is clearly more complex than a simple physiological response to temperature that might be inferred from known geographic ranges.

In addition to being non-destructive, a valuable further advantage to using video systems is the relatively high level of consistency of the sample across a variety of habitats. The efficiency of trawls varies over different habitats, so different types of trawl might be required in order to evaluate overall fish diversity [42], [85]. Baited video systems can be deployed over virtually any kind of habitat, including difficult terrain, such as rough substrata encountered at seamounts [86]. We have used video units with a high success rate over shallow and deep (1200 m) reef systems where trawls would have failed. One might expect that baited cameras will primarily sample effectively only those fish species attracted to bait, or to the lights or activities of other organisms around the bait. Previous studies in shallow water indicate that baited systems can be used to sample a range of trophic groups successfully, including herbivores and omnivores, as well as predatory and scavenging fish species [20]. Nevertheless, the responses of fishes to bait can vary with changes in oceanographic conditions, such as current speed and temperature. Thus deep deployments may attract a different fraction of the ichthyofauna over a similar time frame – a topic worthy of further investigation. Beyond 1500 m, studies have shown that the trophic groups attracted to bait are not only scavengers, but also opportunistic scavengers that are otherwise predators [87], [88]. In this study, both predators and scavengers were observed up to 1200 m, although the primary feeding behaviour of many if not most of the species we recorded at such depths is still largely unknown. Future studies would benefit from comparisons of baited video with other recently developed un-baited video techniques for studying biodiversity and its relationship with habitat characteristics(e.g. [89]).

This study has provided the first characterization of diversity patterns for bait-attracted fish species on continental slopes in New Zealand. This essential observational knowledge of the fine-scale distribution patterns of individual fish species with depth is an imperative primary step towards development of explanatory and predictive ecological models, as well as being fundamental for the implementation of efficient management and conservation strategies for fishery resources.

## Supporting Information

Figure S1 **Number of video samples taken over different types of habitat, as indicated, within each depth stratum.**
(PDF)

Table S1 **Taxa identified from video deployments at three locations in New Zealand waters (ordered phylogenetically).**
(PDF)

Table S2 **Taxa identified from video deployments at three locations in New Zealand waters (ordered alphabetically).**
(PDF)

Table S3 **Taxa identified from video deployments at three locations in New Zealand waters, arranged by their occurrence within each of seven depth strata (50, 100, 300, 500, 700, 900 and 1200 m).**
(PDF)

Video S1 **Selection of video footage showing typical examples of observed fish species attracted to baited remote underwater video systems in New Zealand waters.** Representative footage from three locations (Three Kings Islands, Great Barrier Island and White Island) and seven depth strata are presented (50, 100, 300, 500, 700, 900 and 1200 m).
(MP4)
