# Supplementary material for: Diversity and Composition of Demersal Fishes along a Depth Gradient Assessed by Baited Remote Underwater Stereo-Video
Source: PLoS One. 2012 Oct 31;7(10):e48522. doi: 10.1371/journal.pone.0048522 (PMC3485343; doi:10.1371/journal.pone.0048522)
Supplement: Figure S1 — Number of video samples taken over different types of habitat, as indicated, within each depth stratum. (PDF) [file pone.0048522.s001.pdf]

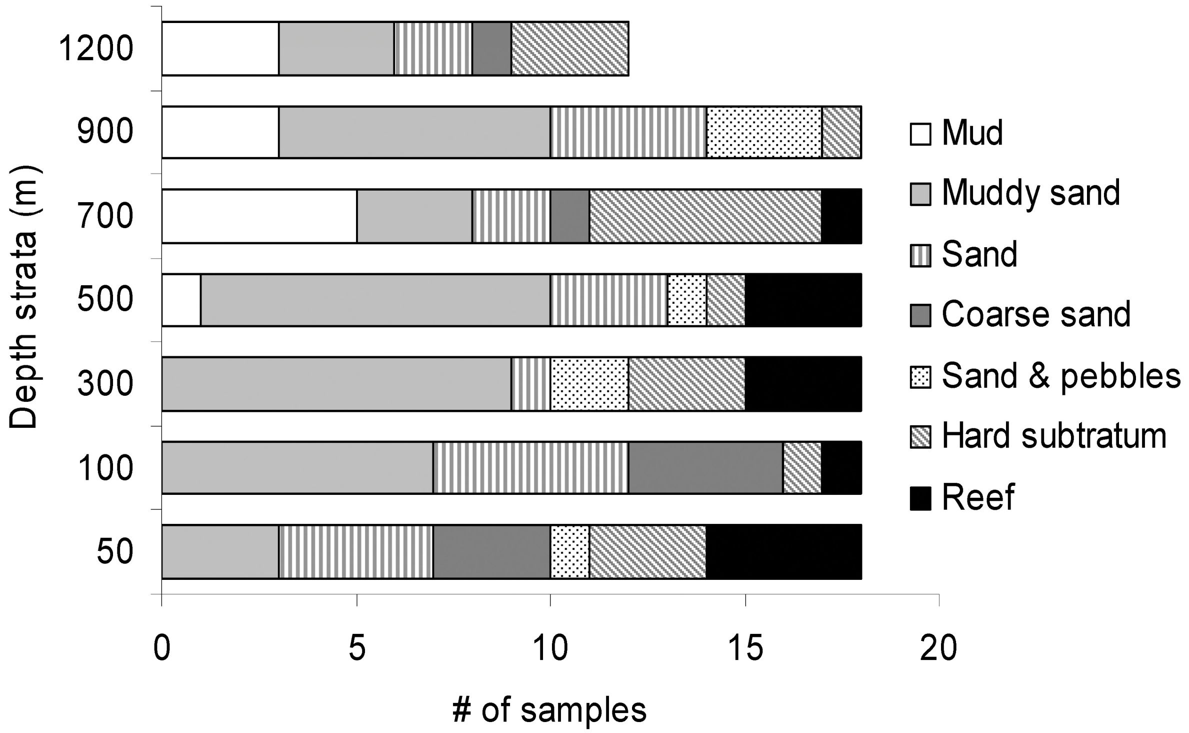

Figure S1. Number of samples showing different types of habitat, as indicated, within each depth stratum, as determined by video images.
