# Supplementary material for: Diversity and Composition of Demersal Fishes along a Depth Gradient Assessed by Baited Remote Underwater Stereo-Video
Source: PLoS One. 2012 Oct 31;7(10):e48522. doi: 10.1371/journal.pone.0048522 (PMC3485343; doi:10.1371/journal.pone.0048522)
Supplement: Table S1 — Taxa identified from video deployments at three locations in New Zealand waters (ordered phylogenetically). (PDF) [file pone.0048522.s002.pdf]

Table S1. Taxa identified from video deployment at three locations in New Zealand waters (ordered phylogenetically). N: total number of individuals observed at one location; MaxN: maximum number of individuals of the same species appearing at the same time at one location, followed by its depth of occurrence; Min/Max depth: minimum/maximum depth at which the species was observed.

TAXA	ENDEMIC	FAMILY	THREE KINGS ISLANDS				GREAT BARRIER ISLANDS				WHITE ISLAND			
			N	MaxN (depth)	Min depth (m)	Max depth (m)	N	MaxN (depth)	Min depth (m)	Max depth (m)	N	MaxN (depth)	Min depth (m)	Max depth (m)
Eptatretus cf.cirrhatus		Myxinidae	4	1 (682)	682	887	53	11 (489)	107	708	57	9 (515)	274	687
Eptatretus sp.2	✓	Myxinidae	33	11 (287)	109	721								
Myxinidae undet.	✓	Myxinidae									1	1 (274)	274	274
Neomyxine sp.1	✓	Myxinidae					24	9 (664)	97	1161	4	2 (914)	715	941
Hexanchus griseus		Hexanchidae	1	1 (721)	721	721								
Odontaspis ferox		Odontaspididae									1	1 (603)	603	603
Isurus oxyrinchus		Lamnidae	2	1 (48)	48	54								
Apristurus sp.		Scyliorhinidae									1	1 (959)	959	959
Cephaloscyllium isabellum	✓	Scyliorhinidae	3	3 (102)	102	102	17	3 (91)	46	538	4	2 (112)	112	313
Parmaturus sp.	✓	Scyliorhinidae	2	1 (1167)	1123	1167	1	1 (1275)	1275	1275				
Galeorhinus galeus		Triakidae	20	3 (102)	48	296	6	1 (62)	62	301				
Gollum attenuatus		Pseudotriakidae	2	1 (489)	489	502	3	2 (538)	282	538				
Cirrhigaleus australis		Squalidae	5	1 (471)	296	701								
Squalus acanthias		Squalidae									1	1 (491)	491	491
Squalus griffini	✓	Squalidae	68	8 (102)	52	721	13	2 (282)	91	538	4	1 (278)	274	336
Squalus sp.		Squalidae	1	1 (666)	666	666								
Squalus sp.5	✓	Squalidae	1	1 (684)	684	684	5	2 (513)	489	708	1	1 (486)	486	486
Centrophorus squamosus		Centrophoridae					5	1 (880)	708	902				
Deania calcea		Centrophoridae	3	1 (937)	721	937	8	2 (664)	664	902	9	2 (914)	687	959
Etmopterus baxteri	✓	Etmopteridae	37	7 (1123)	849	1177	4	2 (1192)	1192	1252				
Etmopterus molleri		Etmopteridae	4	2 (701)	682	701	5	3 (513)	477	513	1	1 (685)	685	685
Etmopterus sp.		Etmopteridae	1	1 (526)	526	526					2	1 (687)	685	687
Centroscymnus owstoni		Somniosidae	15	3 (1148)	887	1177	7	2 (877)	877	1275				
Proscymnodon plunketi		Somniosidae	15	3 (907)	849	1148	1	1 (708)	708	708				
Dalatias licha		Dalatiidae					2	1 (877)	664	877	1	1 (533)	533	533
Dipturus innominatus	✓	Rajidae					6	1 (107)	107	690				
Zearaja nasutus	✓	Rajidae					1	1 (64)	64	64				
Bathyraja shuntovi	✓	Arhynchobatidae					2	1 (680)	680	902				
Dasyatis brevicaudata		Dasyatidae	8	2 (48)	48	102					10	3 (47)	47	104
Dasyatis thetidis		Dasyatidae									1	1 (48)	48	48

TAXA	ENDEMIC	FAMILY	THREE KINGS ISLANDS				GREAT BARRIER ISLANDS				WHITE ISLAND			
			N	MaxN (depth)	Min depth (m)	Max depth (m)	N	MaxN (depth)	Min depth (m)	Max depth (m)	N	MaxN (depth)	Min depth (m)	Max depth (m)
Myliobatis tenuicaudatus	✓	Myliobatidae	2	1 (54)	54	57					1	1 (48)	48	48
Chimaeridae undet.		Chimaeridae					1	1 (708)	708	708				
Hydrolagus sp.		Chimaeridae									1	1 (687)	687	687
Rhinochimaera pacifica		Rhinochimaeridae	1	1 (1148)	1148	1148								
Gymnothorax berndti		Muraenidae									1	1 (52)	52	52
Gymnothorax nubilus		Muraenidae									6	3 (52)	47	52
Gymnothorax prasinus		Muraenidae									3	3 (52)	52	52
Gymnothorax porphyreus		Muraenidae									12	6 (52)	48	112
Bassanago bulbiceps		Congridae	3	1 (937)	701	937	16	3 (690)	474	881	10	2 (914)	603	959
Conger verreauxi		Congridae									1	1 (112)	112	112
Ophisurus serpens		Ophichthidae					1	1 (64)	64	64				
Scolecenchelys castlei		Ophichthidae					4	2 (477)	477	708				
Simenchelys parasitica		Syphobranchidae	12	3 (1177)	849	1177	18	10 (880)	880	1275	1	1 (897)	897	897
Synaphobranchus affinis		Syphobranchidae	74	14 (1148)	849	1177	18	4 (1205)	847	1275	15	3 (914)	685	959
Diastobranchus capensis		Syphobranchidae	32	6 (1129)	907	1177	47	11 (1192)	877	1275	2	1 (890)	880	890
Synaphobranchus sp.		Syphobranchidae									2	1 (687)	687	744
Halosaurus pectoralis		Halosauridae	1	1 (858)	858	858								
Microstomatidae undet.		Microstomatidae					2	2 (286)	286	286				
Sternoptychidae undet.		Sternoptychidae					2	1 (498)	498	513				
Chauliodus sloani		Chauliodontidae					1	1 (1067)	1067	1067				
Stomias sp.		Stomiidae									1	1 (715)	715	715
Alepocephalidae undet.		Alepocephalidae									1	1 (687)	687	687
Alepocephalus australis		Alepocephalidae	1	1 (1148)	1148	1148								
Paraulopus okamurai	✓	Paraulopidae	2	1 (489)	489	502	2	2 (301)	301	301	1	1 (313)	313	313
Myctophidae undet.		Myctophidae					59	48 (477)	477	1192	6	2 (313)	313	959
Scopelosaurus hamiltoni		Notosudidae					1	1 (708)	708	708				
Paralepididae undet.		Paralepididae									2	1 (687)	687	744
Antimora rostrata		Moridae					1	1 (1192)	1192	1192				
Laemonema sp.		Moridae									1	1 (603)	603	603
Lepidion microcephalus		Moridae	2	1 (1115)	1115	1177								
Mora moro		Moridae	20	4 (849)	684	937	17	2 (664)	498	1067	12	2 (533)	491	959
Moridae undet.		Moridae	1	1 (937)	937	937	1	1 (1205)	1205	1205				
Pseudophycis barbata		Moridae									2	1 (274)	274	292

Taxa	Endemic	Family	Three Kings Islands				Great Barrier Islands				White Island			
			N	MaxN (depth)	Min depth (m)	Max depth (m)	N	MaxN (depth)	Min depth (m)	Max depth (m)	N	MaxN (depth)	Min depth (m)	Max depth (m)
Tripterophycis gilchristi		Moridae									1	1 (523)	523	523
Tripterophycis sp.		Moridae					2	1 (498)	498	538				
Macruronus novaezelandiae		Merlucciidae					3	1 (664)	498	664	8	1 (486)	486	880
Genypterus blacodes		Ophidiidae	3	1 (684)	684	721	14	3 (680)	474	859	3	1 (533)	515	715
Caelorinchus acanthiger		Macrouridae					2	1 (1161)	1161	1192				
Caelorinchus sp.		Macrouridae					2	1 (847)	847	881	2	1 (603)	603	880
Coryphaenoides serrulatus		Macrouridae					6	2 (1161)	847	1192	1	1 (880)	880	880
Hymenocephalus sp.		Macrouridae	1	1 (849)	849	849	1	1 (708)	708	708				
Lepidorhynchus denticulatus		Macrouridae									3	1 (491)	491	715
?Lepidorhynchus sp.		Macrouridae					1	1 (708)	708	708				
Macrouridae undet.		Macrouridae	5	1 (887)	513	1129	8	1 (880)	708	1252	5	1 (687)	685	941
Trachyrincus aphyodes	✓	Macrouridae					2	1 (877)	877	1192				
Bathygadus cottoides		Bathygadidae					1	1 (1205)	1205	1205				
Gadomus aoteanus		Bathygadidae	3	1 (1148)	1129	1177	4	1 (881)	881	1275	2	1 (941)	880	941
Hoplostethus mediterraneus		Trachichthyidae	2	1 (858)	849	858	6	3 (513)	498	680				
Hoplostethus sp.		Trachichthyidae									2	2 (685)	685	685
Beryx decadactylus		Berycidae									1	1 (491)	491	491
Centroberyx affinis		Berycidae	28	11 (105)	52	112					32	29 (112)	52	112
Capromimus abbreviatus	✓	Zeniontidae					1	1 (498)	498	498				
Zenion leptolepis		Zeniontidae					1	1 (301)	301	301				
Zeidae undet.		Zeidae									1	1 (491)	491	491
Zeus faber		Zeidae					2	2 (91)	91	91				
Cyttus novaezealandiae		Cyttidae									1	1 (486)	486	486
Oreosomatidae undet.		Oreosomatidae					1	1 (1161)	1161	1161				
Centriscops humerosus		Macroramphosidae					3	2 (498)	477	498	3	2 (491)	491	515
Helicolenus percoides		Scorpaenidae									7	4 (274)	274	313
Helicolenus sp.		Scorpaenidae	14	4 (344)	106	701	14	3 (62)	62	538	19	4 (491)	290	715
Scorpaena sp.		Scorpaenidae					2	2 (62)	62	62	3	2 (52)	52	112
Chelidonichthys kumu		Triglidae					8	3 (39)	39	101				
Pterygotrigla andertoni		Triglidae	1	1 (296)	296	296	1	1 (301)	301	301	2	1 (55)	55	292
Caesioperca lepidoptera		Serranidae	197	116 (52)	52	109									

Taxa	Endemic	Family	Three Kings Islands				Great Barrier Islands				White Island			
			N	MaxN (depth)	Min depth (m)	Max depth (m)	N	MaxN (depth)	Min depth (m)	Max depth (m)	N	MaxN (depth)	Min depth (m)	Max depth (m)
Caprodon longimanus		Serranidae	367	129 (57)	48	109	14	13 (49)	49	62	33	25 (55)	55	112
Hypoplectrodes sp.B	✓	Serranidae	1	1 (52)	52	52	1	1 (62)	62	62				
Lepidoperca inornata	✓	Serranidae	2	2 (287)	287	287								
Lepidoperca sp.		Serranidae	1	1 (287)	287	287								
Plectranthias maculicauda		Serranidae	6	2 (287)	106	489	1	1 (91)	91	91				
Callanthias australis		Callanthiidae	2	2 (57)	57	57								
Callanthias sp.		Callanthiidae					1	1 (62)	62	62				
Polyprion americanus		Polyprionidae	18	4 (344)	109	699					1	1 (292)	292	292
Polyprion oxygeneios		Polyprionidae	7	3 (109)	57	112	2	1 (301)	91	301				
Pseudocaranx georgianus		Carangidae	7	1 (48)	48	106	14	9 (64)	46	107	29	17 (47)	47	55
Pseudocaranx sp.*dentex*		Carangidae									2	2 (47)	47	47
Seriola lalandi		Carangidae	34	7 (54)	48	287	27	14 (62)	39	101	15	4 (48)	47	112
Arripis xylabion		Arripidae					2	1 (107)	49	107				
Pagrus auratus		Sparidae	29	13 (48)	48	102	65	14 (46)	39	107	2	2 (48)	48	48
Upeneichthys lineatus		Mullidae					1	1 (62)	62	62	1	1 (51)	51	51
Amphichaetodon howensis		Chaetodontidae									2	2 (52)	52	52
Chromis dispila	✓	Pomacentridae									20	20 (52)	52	52
Chromis sp.		Pomacentridae	3	2 (54)	54	63								
Nemadactylus douglasii		Cheilodactylidae	6	2 (48)	48	105	2	2 (49)	49	49	7	4 (55)	47	55
Nemadactylus macropterus		Cheilodactylidae	18	8 (105)	48	109	34	15 (107)	64	301	72	24 (110)	52	336
Nemadactylus n.sp.		Cheilodactylidae	29	14 (109)	65	287	1	1 (46)	46	46				
Latridopsis ciliaris		Latridae									1	1 (48)	48	48
Latridopsis forsteri		Latridae									1	1 (55)	55	55
Bodianus unimaculatus		Labridae					6	4 (62)	49	91	10	5 (52)	52	99
Coris sandeyeri		Labridae									1	1 (52)	52	52
Notolabrus cinctus	✓	Labridae	1	1 (109)	109	109								
Notolabrus fucicola		Labridae	1	1 (57)	57	57								
Pseudolabrus miles	✓	Labridae	77	37 (57)	48	109	2	1 (62)	49	62				
Suezichthys aylingi		Labridae	192	59 (65)	48	109								
Parapercis binivirgata		Pinguipedidae	3	2 (106)	106	112								
Parapercis colias	✓	Pinguipedidae	26	8 (65)	52	112	7	5 (49)	49	62				
Parapercis gilliesi	✓	Pinguipedidae	8	3 (296)	105	296								
Forsterygion flavonigrum	✓	Tripterygiidae	1	1 (54)	54	54	3	2 (62)	62	91				

Taxa	Endemic	Family	Three Kings Islands				Great Barrier Islands				White Island			
			N	MaxN (depth)	Min depth (m)	Max depth (m)	N	MaxN (depth)	Min depth (m)	Max depth (m)	N	MaxN (depth)	Min depth (m)	Max depth (m)
Gempylidae undet.		Gempylidae					1	1 (708)	708	708	1	1 (687)	687	687
Rexea solandri		Gempylidae	1	1 (701)	701	701	8	1 (282)	280	664	8	1 (486)	274	959
Ruvettus pretiosus		Gempylidae					5	2 (902)	680	1067	3	1 (941)	880	941
Thyrsites atun		Gempylidae	3	2 (106)	106	109	3	2 (49)	49	103				
Benthodesmus sp.		Trichiuridae					2	1 (477)	477	498	2	2 (515)	515	515
Lepidopus caudatus		Trichiuridae					1	1 (62)	62	62				
Hyperoglyphe antarctica		Centrolophidae	27	8 (344)	296	721	3	2 (538)	538	680	5	1 (486)	313	699
Parika scaber		Monacanthidae	30	10 (65)	48	112	1	1 (62)	62	62	3	1 (47)	47	55
Canthigaster callisterna		Tetraodontidae									2	1 (52)	51	52
Tragulichthys pilatus		Diodontidae	2	1 (54)	54	65								
