# Supplementary material for: Diversity and Composition of Demersal Fishes along a Depth Gradient Assessed by Baited Remote Underwater Stereo-Video
Source: PLoS One. 2012 Oct 31;7(10):e48522. doi: 10.1371/journal.pone.0048522 (PMC3485343; doi:10.1371/journal.pone.0048522)
Supplement: Table S3 — Taxa identified from video deployments at three locations in New Zealand waters, arranged by their occurrence within each of seven depth strata (50, 100, 300, 500, 700, 900 and 1200 m). (PDF) [file pone.0048522.s004.pdf]

**Table S3.** Taxa identified from video deployment at three locations in New Zealand waters, arranged by their occurrence within seven depth strata (50, 100, 300, 500, 700, 900 and 1200 m). Depth range and distribution from this study are also given. TKI: Three Kings Islands, GBI: Great Barrier Island, WI: White Island.

| TAXA | FAMILY | ORDER | DEPTH RANGE (m) | DISTRIBUTION |
|---|---|---|---|---|
| **50m depth strata** | | | | |
| CHONDRICHTHYES (N=8) | | | | |
| *Cephaloscyllium isabellum* | Scyliorhinidae | Carcharhiniformes | 46 - 538 | TKI, GBI, WI |
| *Dasyatis brevicaudata* | Dasyatidae | Rajiformes | 47 - 104 | TKI WI |
| *Dasyatis thetidis* | Dasyatidae | Rajiformes | 48 | WI |
| *Galeorhinus galeus* | Triakidae | Carcharhiniformes | 48 - 301 | TKI, GBI |
| *Isurus oxyrinchus* | Lamnidae | Lamniformes | 48 - 54 | TKI |
| *Myliobatis tenuicaudatus* | Myliobatidae | Rajiformes | 48 - 57 | TKI WI |
| *Squalus griffini* | Squalidae | Squaliformes | 52 - 721 | TKI, GBI, WI |
| *Zearaja nasutus* | Rajidae | Rajiformes | 64 | GBI |
| OSTEICHTHYES (N=42) | | | | |
| *Amphichaetodon howensis* | Chaetodontidae | Perciformes | 52 | WI |
| *Arripis xylabion* | Arripidae | Perciformes | 49 - 107 | GBI |
| *Bodianus unimaculatus* | Labridae | Perciformes | 49 - 99 | GBI, WI |
| *Caesioperca lepidoptera* | Serranidae | Perciformes | 52 - 109 | TKI |
| *Callanthias australis* | Callanthiidae | Perciformes | 57 | TKI |
| *Callanthias* sp. | Callanthiidae | Perciformes | 62 | GBI |
| *Canthigaster callisterna* | Tetraodontidae | Tetraodontiformes | 51 - 52 | WI |
| *Caprodon longimanus* | Serranidae | Perciformes | 48 - 112 | TKI, GBI, WI |
| *Centroberyx affinis* | Berycidae | Beryciformes | 52 - 112 | TKI WI |
| *Chelidonichthys kumu* | Triglidae | Scorpaeniformes | 39 - 101 | GBI |
| *Chromis dispila* | Pomacentridae | Perciformes | 52 | WI |
| *Chromis* sp. | Pomacentridae | Perciformes | 54 - 63 | TKI |
| *Coris sandeyeri* | Labridae | Perciformes | 52 | WI |
| *Forsterygion flavonigrum* | Tripterygiidae | Perciformes | 54 - 91 | TKI, GBI |
| *Gymnothorax berndti* | Muraenidae | Anguilliformes | 52 | WI |
| *Gymnothorax nubilus* | Muraenidae | Anguilliformes | 47 - 52 | WI |
| *Gymnothorax prasinus* | Muraenidae | Anguilliformes | 52 | WI |
| *Gymnothorax prophyreus* | Muraenidae | Anguilliformes | 48 - 112 | WI |
| *Helicolenus* sp. | Scorpaenidae | Scorpaeniformes | 62 - 715 | TKI, GBI, WI |
| *Hypoplectrodes* sp.B | Serranidae | Perciformes | 52 - 62 | TKI, GBI |
| *Latridopsis ciliaris* | Latridae | Perciformes | 48 | WI |
| *Latridopsis forsteri* | Latridae | Perciformes | 55 | WI |
| *Lepidopus caudatus* | Trichiuridae | Perciformes | 62 | GBI |
| *Nemadactylus douglasii* | Cheilodactylidae | Perciformes | 47 - 105 | TKI, GBI, WI |
| *Nemadactylus macropterus* | Cheilodactylidae | Perciformes | 48 - 336 | TKI, GBI, WI |
| *Nemadactylus* n.sp. | Cheilodactylidae | Perciformes | 46 - 287 | TKI, GBI |
| *Notolabrus fucicola* | Labridae | Perciformes | 57 | TKI |
| *Ophisurus serpens* | Ophichthidae | Anguilliformes | 64 | GBI |
| *Pagrus auratus* | Sparidae | Perciformes | 39 - 107 | TKI, GBI, WI |
| *Parapercis colias* | Pinguipedidae | Perciformes | 49 - 112 | TKI, GBI |
| *Parika scaber* | Monacanthidae | Tetraodontiformes | 47 - 112 | TKI, GBI, WI |
| *Polyprion oxygeneios* | Polyprionidae | Perciformes | 57 - 301 | TKI, GBI |
| *Pseudocaranx georgianus* | Carangidae | Perciformes | 46 - 107 | TKI, GBI, WI |
| *Pseudocaranx* sp.dentex | Carangidae | Perciformes | 47 | WI |
| *Pseudolabrus miles* | Labridae | Perciformes | 48 - 109 | TKI, GBI |
| *Pterygotrigla andertoni* | Triglidae | Scorpaeniformes | 55 - 301 | TKI, GBI, WI |
| *Scorpaena* sp. | Scorpaenidae | Scorpaeniformes | 52 - 112 | GBI, WI |
| *Seriola lalandi* | Carangidae | Perciformes | 39 - 287 | TKI, GBI, WI |
| *Suezichthys aylingi* | Labridae | Perciformes | 48 - 109 | TKI |
| *Thyrsites atun* | Gempylidae | Perciformes | 49 - 109 | TKI, GBI |
| *Tragulichthys pilatus* | Diodontidae | Tetraodontiformes | 54 - 65 | TKI |
| *Upeneichthys lineatus* | Mullidae | Perciformes | 51 - 62 | GBI, WI |

| TAXA | FAMILY | ORDER | DEPTH RANGE (m) | DISTRIBUTION |
|---|---|---|---|---|
| **100m depth strata** | | | | |
| MYXINI (N=3) | | | | |
| *Eptatretus cf.cirrhatus* | Myxinidae | Myxiniformes | 107 - 887 | TKI, GBI, WI |
| *Eptatretus* sp.2 | Myxinidae | Myxiniformes | 109 - 721 | TKI |
| *Neomyxine* sp.1 | Myxinidae | Myxiniformes | 97 - 1161 | GBI, WI |
| CHONDRICHTHYES (N=5) | | | | |
| *Cephaloscyllium isabellum* | Scyliorhinidae | Carcharhiniformes | 46 - 538 | TKI, GBI, WI |
| *Dasyatis brevicaudata* | Dasyatidae | Rajiformes | 47 - 104 | TKI WI |
| *Dipturus innominatus* | Rajidae | Rajiformes | 107 - 690 | GBI |
| *Galeorhinus galeus* | Triakidae | Carcharhiniformes | 48 - 301 | TKI, GBI |
| *Squalus griffini* | Squalidae | Squaliformes | 52 - 721 | TKI, GBI, WI |
| OSTEICHTHYES (N=29) | | | | |
| *Arripis xylabion* | Arripidae | Perciformes | 49 - 107 | GBI |
| *Bodianus unimaculatus* | Labridae | Perciformes | 49 - 99 | GBI, WI |
| *Caesioperca lepidoptera* | Serranidae | Perciformes | 52 - 109 | TKI |
| *Caprodon longimanus* | Serranidae | Perciformes | 48 - 112 | TKI, GBI, WI |
| *Centroberyx affinis* | Berycidae | Beryciformes | 52 - 112 | TKI WI |
| *Chelidonichthys kumu* | Triglidae | Scorpaeniformes | 39 - 101 | GBI |
| *Conger verreauxi* | Congridae | Anguilliformes | 112 | WI |
| *Forsterygion flavonigrum* | Tripterygiidae | Perciformes | 54 - 91 | TKI, GBI |
| *Gymnothorax prophyreus* | Muraenidae | Anguilliformes | 48 - 112 | WI |
| *Helicolenus* sp. | Scorpaenidae | Scorpaeniformes | 62 - 715 | TKI, GBI, WI |
| *Nemadactylus douglasii* | Cheilodactylidae | Perciformes | 47 - 105 | TKI, GBI, WI |
| *Nemadactylus macropterus* | Cheilodactylidae | Perciformes | 48 - 336 | TKI, GBI, WI |
| *Nemadactylus* n.sp. | Cheilodactylidae | Perciformes | 46 - 287 | TKI, GBI |
| *Notolabrus cinctus* | Labridae | Perciformes | 109 | TKI |
| *Pagrus auratus* | Sparidae | Perciformes | 39 - 107 | TKI, GBI, WI |
| *Parapercis binivirgata* | Pinguipedidae | Perciformes | 106 - 112 | TKI |
| *Parapercis colias* | Pinguipedidae | Perciformes | 49 - 112 | TKI, GBI |
| *Parapercis gilliesi* | Pinguipedidae | Perciformes | 105 - 296 | TKI |
| *Parika scaber* | Monacanthidae | Tetraodontiformes | 47 - 112 | TKI, GBI, WI |
| *Plectranthias maculicauda* | Serranidae | Perciformes | 91 - 489 | TKI, GBI |
| *Polyprion americanus* | Polyprionidae | Perciformes | 109 - 699 | TKI WI |
| *Polyprion oxygeneios* | Polyprionidae | Perciformes | 57 - 301 | TKI, GBI |
| *Pseudocaranx georgianus* | Carangidae | Perciformes | 46 - 107 | TKI, GBI, WI |
| *Pseudolabrus miles* | Labridae | Perciformes | 48 - 109 | TKI, GBI |
| *Scorpaena* sp. | Scorpaenidae | Scorpaeniformes | 52 - 112 | GBI, WI |
| *Seriola lalandi* | Carangidae | Perciformes | 39 - 287 | TKI, GBI, WI |
| *Suezichthys aylingi* | Labridae | Perciformes | 48 - 109 | TKI |
| *Thyrsites atun* | Gempylidae | Perciformes | 49 - 109 | TKI, GBI |
| *Zeus faber* | Zeidae | Zeiformes | 91 | GBI |
| **300m depth strata** | | | | |
| MYXINI (N=4) | | | | |
| *Eptatretus cf.cirrhatus* | Myxinidae | Myxiniformes | 107 - 887 | TKI, GBI, WI |
| *Eptatretus* sp.2 | Myxinidae | Myxiniformes | 109 - 721 | TKI |
| Myxinidae undet. | Myxinidae | Myxiniformes | 274 | WI |
| *Neomyxine* sp.1 | Myxinidae | Myxiniformes | 97 - 1161 | GBI, WI |
| CHONDRICHTHYES (N=5) | | | | |
| *Cephaloscyllium isabellum* | Scyliorhinidae | Carcharhiniformes | 46 - 538 | TKI, GBI, WI |
| *Cirrhigaleus australis* | Squalidae | Squaliformes | 296 - 701 | TKI |
| *Galeorhinus galeus* | Triakidae | Carcharhiniformes | 48 - 301 | TKI, GBI |
| *Gollum attenuatus* | Pseudotriakidae | Carcharhiniformes | 282 - 538 | TKI, GBI |
| *Squalus griffini* | Squalidae | Squaliformes | 52 - 721 | TKI, GBI, WI |

| Taxa | Family | Order | Depth range (m) | Distribution |
|---|---|---|---|---|
| Osteichthyes (N=19) | | | | |
| *Helicolenus percoides* | Scorpaenidae | Scorpaeniformes | 274 - 313 | WI |
| *Helicolenus* sp. | Scorpaenidae | Scorpaeniformes | 62 - 715 | TKI, GBI, WI |
| *Hyperoglyphe antarctica* | Centrolophidae | Perciformes | 296 - 721 | TKI, GBI, WI |
| *Lepidoperca inornata* | Serranidae | Perciformes | 287 | TKI |
| *Lepidoperca* sp. | Serranidae | Perciformes | 287 | TKI |
| Microstomatidae undet. | Microstomatidae | Osmeriformes | 286 | GBI |
| Myctophidae undet. | Myctophidae | Myctophiformes | 313 - 1192 | GBI, WI |
| *Nemadactylus macropterus* | Cheilodactylidae | Perciformes | 48 - 336 | TKI, GBI, WI |
| *Nemadactylus* n.sp. | Cheilodactylidae | Perciformes | 46 - 287 | TKI, GBI |
| *Parapercis gilliesi* | Pinguipedidae | Perciformes | 105 - 296 | TKI |
| *Paraulopus okamurai* | Paraulopidae | Aulopiformes | 301 - 502 | TKI, GBI, WI |
| *Plectranthias maculicauda* | Serranidae | Perciformes | 91 - 489 | TKI, GBI |
| *Polyprion americanus* | Polyprionidae | Perciformes | 109 - 699 | TKI WI |
| *Polyprion oxygeneios* | Polyprionidae | Perciformes | 57 - 301 | TKI, GBI |
| *Pseudophycis barbata* | Moridae | Gadiformes | 274 - 292 | WI |
| *Pterygotrigla andertoni* | Triglidae | Scorpaeniformes | 55 - 301 | TKI, GBI, WI |
| *Rexea solandri* | Gempylidae | Perciformes | 274 - 959 | TKI, GBI, WI |
| *Seriola lalandi* | Carangidae | Perciformes | 39 - 287 | TKI, GBI, WI |
| *Zenion leptolepis* | Zenionidae | Zeiformes | 301 | GBI |
| **500m depth strata** | | | | |
| Myxini (N=2) | | | | |
| *Eptatretus cf.cirrhatus* | Myxinidae | Myxiniformes | 107 - 887 | TKI, GBI, WI |
| *Eptatretus* sp.2 | Myxinidae | Myxiniformes | 109 - 721 | TKI |
| Chondrichthyes (N=10) | | | | |
| *Cephaloscyllium isabellum* | Scyliorhinidae | Carcharhiniformes | 46 - 538 | TKI, GBI, WI |
| *Cirrhigaleus australis* | Squalidae | Squaliformes | 296 - 701 | TKI |
| *Dalatias licha* | Dalatiidae | Squaliformes | 533 - 877 | GBI, WI |
| *Dipturus innominatus* | Rajidae | Rajiformes | 107 - 690 | GBI |
| *Etmopterus molleri* | Etmopteridae | Squaliformes | 477 - 701 | TKI, GBI, WI |
| *Etmopterus* sp. | Etmopteridae | Squaliformes | 526 - 687 | TKI WI |
| *Gollum attenuatus* | Pseudotriakidae | Carcharhiniformes | 282 - 538 | TKI, GBI |
| *Squalus acanthias* | Squalidae | Squaliformes | 491 | WI |
| *Squalus griffini* | Squalidae | Squaliformes | 52 - 721 | TKI, GBI, WI |
| *Squalus* sp.5 | Squalidae | Squaliformes | 486 - 708 | TKI, GBI, WI |
| Osteichthyes (N=24) | | | | |
| *Bassanago bulbiceps* | Congridae | Anguilliformes | 474 - 959 | TKI, GBI, WI |
| *Benthodesmus* sp. | Trichiuridae | Perciformes | 477 - 515 | GBI, WI |
| *Beryx decadactylus* | Berycidae | Beryciformes | 491 | WI |
| *Capromimus abbreviatus* | Zeniontidae | Zeiformes | 498 | GBI |
| *Centriscops humerosus* | Macroramphosidae | Syngnathiformes | 477 - 515 | GBI, WI |
| *Cyttus novaezealandiae* | Cyttidae | Zeiformes | 486 | WI |
| *Genypterus blacodes* | Ophidiidae | Ophidiiformes | 474 - 859 | TKI, GBI, WI |
| *Helicolenus* sp. | Scorpaenidae | Scorpaeniformes | 62 - 715 | TKI, GBI, WI |
| *Hoplostethus mediterraneus* | Trachichthyidae | Beryciformes | 498 - 858 | TKI, GBI |
| *Hyperoglyphe antarctica* | Centrolophidae | Perciformes | 296 - 721 | TKI, GBI, WI |
| *Lepidorhynchus denticulatus* | Macrouridae | Gadiformes | 491 - 715 | WI |
| Macrouridae undet. | Macrouridae | Gadiformes | 513 - 1252 | TKI, GBI, WI |
| *Macruronus novaezelandiae* | Merlucciidae | Gadiformes | 486 - 880 | GBI, WI |
| *Mora moro* | Moridae | Gadiformes | 491 - 1067 | TKI, GBI, WI |
| Myctophidae undet. | Myctophidae | Myctophiformes | 313 - 1192 | GBI, WI |
| *Paraulopus okamurai* | Paraulopidae | Aulopiformes | 301 - 502 | TKI, GBI, WI |
| *Plectranthias maculicauda* | Serranidae | Perciformes | 91 - 489 | TKI, GBI |
| *Polyprion americanus* | Polyprionidae | Perciformes | 109 - 699 | TKI WI |

| Taxa | Family | Order | Depth range (m) | Distribution |
|---|---|---|---|---|
| *Rexea solandri* | Gempylidae | Perciformes | 274 - 959 | TKI, GBI, WI |
| *Scolecenchelys castlei* | Ophichthidae | Anguilliformes | 477 - 708 | GBI |
| Sternoptychidae undet. | Sternoptychidae | Stomiiformes | 498 - 513 | GBI |
| *Tripterophycis gilchristi* | Moridae | Gadiformes | 523 | WI |
| *Tripterophycis* sp. | Moridae | Gadiformes | 498 - 538 | GBI |
| Zeidae undet. | Zeidae | Zeiformes | 491 | WI |
| **700m depth strata** | | | | |
| Myxini (N=3) | | | | |
| *Eptatretus cf.cirrhatus* | Myxinidae | Myxiniformes | 107 - 887 | TKI, GBI, WI |
| *Eptatretus* sp.2 | Myxinidae | Myxiniformes | 109 - 721 | TKI |
| *Neomyxine* sp.1 | Myxinidae | Myxiniformes | 97 - 1161 | GBI, WI |
| Chondrichthyes (N=16) | | | | |
| *Bathyraja shuntovi* | Arhynchobatidae | Rajiformes | 680 - 902 | GBI |
| *Centrophorus squamosus* | Centrophoridae | Squaliformes | 708 - 902 | GBI |
| Chimaeridae undet. | Chimaeridae | Chimaeriformes | 708 | GBI |
| *Cirrhigaleus australis* | Squalidae | Squaliformes | 296 - 701 | TKI |
| *Dalatias licha* | Dalatiidae | Squaliformes | 533 - 877 | GBI, WI |
| *Deania calcea* | Centrophoridae | Squaliformes | 664 - 959 | TKI, GBI, WI |
| *Dipturus innominatus* | Rajidae | Rajiformes | 107 - 690 | GBI |
| *Etmopterus molleri* | Etmopteridae | Squaliformes | 477 - 701 | TKI, GBI, WI |
| *Etmopterus* sp. | Etmopteridae | Squaliformes | 526 - 687 | TKI WI |
| *Hexanchus griseus* | Hexanchidae | Hexanchiformes | 721 | TKI |
| *Hydrolagus* sp. | Chimaeridae | Chimaeriformes | 687 | WI |
| *Odontaspis ferox* | Odontaspididae | Lamniformes | 603 | WI |
| *Proscymnodon plunketi* | Somniosidae | Squaliformes | 708 - 1148 | TKI, GBI |
| *Squalus griffini* | Squalidae | Squaliformes | 52 - 721 | TKI, GBI, WI |
| *Squalus* sp. | Squalidae | Squaliformes | 666 | TKI |
| *Squalus* sp.5 | Squalidae | Squaliformes | 486 - 708 | TKI, GBI, WI |
| Osteichthyes (N=26) | | | | |
| Alepocephalidae undet. | Alepocephalidae | Osmeriformes | 687 | WI |
| *Bassanago bulbiceps* | Congridae | Anguilliformes | 474 - 959 | TKI, GBI, WI |
| *Caelorinchus* sp. | Macrouridae | Gadiformes | 603 - 881 | GBI, WI |
| Gempylidae undet. | Gempylidae | Perciformes | 687 - 708 | GBI, WI |
| *Genypterus blacodes* | Ophidiidae | Ophidiiformes | 474 - 859 | TKI, GBI, WI |
| *Helicolenus* sp. | Scorpaenidae | Scorpaeniformes | 62 - 715 | TKI, GBI, WI |
| *Hoplostethus mediterraneus* | Trachichthyidae | Beryciformes | 498 - 858 | TKI, GBI |
| *Hoplostethus* sp. | Trachichthyidae | Beryciformes | 685 | WI |
| *Hymenocephalus* sp. | Macrouridae | Gadiformes | 708 - 849 | TKI, GBI |
| *Hyperoglyphe antarctica* | Centrolophidae | Perciformes | 296 - 721 | TKI, GBI, WI |
| *Laemonema* sp. | Moridae | Gadiformes | 603 | WI |
| *Lepidorhynchus denticulatus* | Macrouridae | Gadiformes | 491 - 715 | WI |
| *?Lepidorhynchus* sp. | Macrouridae | Gadiformes | 708 | GBI |
| Macrouridae undet. | Macrouridae | Gadiformes | 513 - 1252 | TKI, GBI, WI |
| *Macruronus novaezelandiae* | Merlucciidae | Gadiformes | 486 - 880 | GBI, WI |
| *Mora moro* | Moridae | Gadiformes | 491 - 1067 | TKI, GBI, WI |
| Myctophidae undet. | Myctophidae | Myctophiformes | 313 - 1192 | GBI, WI |
| Paralepididae undet. | Paralepididae | Aulopiformes | 687 - 744 | WI |
| *Polyprion americanus* | Polyprionidae | Perciformes | 109 - 699 | TKI WI |
| *Rexea solandri* | Gempylidae | Perciformes | 274 - 959 | TKI, GBI, WI |
| *Ruvettus pretiosus* | Gempylidae | Perciformes | 680 - 1067 | GBI, WI |
| *Scolecenchelys castlei* | Ophichthidae | Anguilliformes | 477 - 708 | GBI |
| *Scopelosaurus hamiltoni* | Notosudidae | Aulopiformes | 708 | GBI |
| *Stomias* sp. | Stomiidae | Stomiiformes | 715 | WI |
| *Synaphobranchus affinis* | Synaphobranchidae | Anguilliformes | 685 - 1275 | TKI, GBI, WI |
| *Synaphobranchus* sp. | Synaphobranchidae | Anguilliformes | 687 - 744 | WI |

| TAXA | FAMILY | ORDER | DEPTH RANGE (m) | DISTRIBUTION |
|---|---|---|---|---|
| *Apristurus* sp. | Scyliorhinidae | Carcharhiniformes | 959 | WI |
| **900m depth strata** | | | | |
| MYXINI (N=2) | | | | |
| *Eptatretus cf.cirrhatus* | Myxinidae | Myxiniformes | 107 - 887 | TKI, GBI, WI |
| *Neomyxine* sp.1 | Myxinidae | Myxiniformes | 97 - 1161 | GBI, WI |
| CHONDRICHTHYES (N=8) | | | | |
| *Bathyraja shuntovi* | Arhynchobatidae | Rajiformes | 680 - 902 | GBI |
| *Centrophorus squamosus* | Centrophoridae | Squaliformes | 708 - 902 | GBI |
| *Centroscymnus owstoni* | Somniosidae | Squaliformes | 877 - 1275 | TKI, GBI |
| *Dalatias licha* | Dalatiidae | Squaliformes | 533 - 877 | GBI, WI |
| *Deania calcea* | Centrophoridae | Squaliformes | 664 - 959 | TKI, GBI, WI |
| *Diastobranchus capensis* | Centrophoridae | Squaliformes | 877 - 1275 | TKI, GBI, WI |
| *Etmopterus baxteri* | Etmopteridae | Squaliformes | 849 - 1252 | TKI, GBI |
| *Proscymnodon plunketi* | Somniosidae | Squaliformes | 708 - 1148 | TKI, GBI |
| OSTEICHTHYES (N=18) | | | | |
| *Bassanago bulbiceps* | Congridae | Anguilliformes | 474 - 959 | TKI, GBI, WI |
| *Caelorinchus* sp. | Macrouridae | Gadiformes | 603 - 881 | GBI, WI |
| *Coryphaenoides serrulatus* | Macrouridae | Gadiformes | 847 - 1192 | GBI, WI |
| *Gadomus aoteanus* | Bathygadidae | Gadiformes | 880 - 1275 | TKI, GBI, WI |
| *Genypterus blacodes* | Ophidiidae | Ophidiiformes | 474 - 859 | TKI, GBI, WI |
| *Halosaurus pectoralis* | Halosauridae | Notacanthiformes | 858 | TKI |
| *Hoplostethus mediterraneus* | Trachichthyidae | Beryciformes | 498 - 858 | TKI, GBI |
| *Hymenocephalus* sp. | Macrouridae | Gadiformes | 708 - 849 | TKI, GBI |
| Macrouridae undet. | Macrouridae | Gadiformes | 513 - 1252 | TKI, GBI, WI |
| *Macruronus novaezelandiae* | Merlucciidae | Gadiformes | 486 - 880 | GBI, WI |
| *Mora moro* | Moridae | Gadiformes | 491 - 1067 | TKI, GBI, WI |
| Moridae undet. | Moridae | Gadiformes | 937 - 1205 | TKI, GBI |
| Myctophidae undet. | Myctophidae | Myctophiformes | 313 - 1192 | GBI, WI |
| *Rexea solandri* | Gempylidae | Perciformes | 274 - 959 | TKI, GBI, WI |
| *Ruvettus pretiosus* | Gempylidae | Perciformes | 680 - 1067 | GBI, WI |
| *Simenchelys parasitica* | Synaphobranchidae | Anguilliformes | 849 - 1275 | TKI, GBI, WI |
| *Synaphobranchus affinis* | Synaphobranchidae | Anguilliformes | 685 - 1275 | TKI, GBI, WI |
| *Trachyrincus aphyodes* | Macrouridae | Gadiformes | 877 - 1192 | GBI |
| **1200m depth strata** | | | | |
| MYXINI (N=1) | | | | |
| *Neomyxine* sp.1 | Myxinidae | Myxiniformes | 97 - 1161 | GBI, WI |
| CHONDRICHTHYES (N=6) | | | | |
| *Centroscymnus owstoni* | Somniosidae | Squaliformes | 877 - 1275 | TKI, GBI |
| *Diastobranchus capensis* | Centrophoridae | Squaliformes | 877 - 1275 | TKI, GBI, WI |
| *Etmopterus baxteri* | Etmopteridae | Squaliformes | 849 - 1252 | TKI, GBI |
| *Parmaturus* sp. | Scyliorhinidae | Carcharhiniformes | 1123 - 1275 | TKI, GBI |
| *Proscymnodon plunketi* | Somniosidae | Squaliformes | 708 - 1148 | TKI, GBI |
| *Rhinochimaera pacifica* | Rhinochimaeridae | Chimaeriformes | 1148 | TKI |
| OSTEICHTHYES (N=17) | | | | |
| *Alepocephalus australis* | Alepocephalidae | Osmeriformes | 1148 | TKI |
| *Antimora rostrata* | Moridae | Gadiformes | 1192 | GBI |
| *Bathygadus cottoides* | Bathygadidae | Gadiformes | 1205 | GBI |
| *Caelorinchus acanthiger* | Macrouridae | Gadiformes | 1161 - 1192 | GBI |
| *Chauliodus sloani* | Stomiidae | Stomiiformes | 1067 | GBI |
| *Coryphaenoides serrulatus* | Macrouridae | Gadiformes | 847 - 1192 | GBI, WI |
| *Gadomus aoteanus* | Bathygadidae | Gadiformes | 880 - 1275 | TKI, GBI, WI |

| Taxa | Family | Order | Depth range (m) | Distribution |
|---|---|---|---|---|
| *Lepidion microcephalus* | Moridae | Gadiformes | 1115 - 1177 | TKI |
| Macrouridae undet. | Macrouridae | Gadiformes | 513 - 1252 | TKI, GBI, WI |
| *Mora moro* | Moridae | Gadiformes | 491 - 1067 | TKI, GBI, WI |
| Moridae undet. | Moridae | Gadiformes | 937 - 1205 | TKI, GBI |
| Myctophidae undet. | Myctophidae | Myctophiformes | 313 - 1192 | GBI, WI |
| Oreosomatidae undet. | Oreosomatidae | Zeiformes | 1161 | GBI |
| *Ruvettus pretiosus* | Gempylidae | Perciformes | 680 - 1067 | GBI, WI |
| *Simenchelys parasitica* | Synaphobranchidae | Anguilliformes | 849 - 1275 | TKI, GBI, WI |
| *Synaphobranchus affinis* | Synaphobranchidae | Anguilliformes | 685 - 1275 | TKI, GBI, WI |
| *Trachyrincus aphyodes* | Macrouridae | Gadiformes | 877 - 1192 | GBI |
